# CaMKII-mediated Beclin 1 phosphorylation regulates autophagy that promotes degradation of Id and neuroblastoma cell differentiation

**DOI:** 10.1038/s41467-017-01272-2

**Published:** 2017-10-27

**Authors:** Xuan Li, Xiao-Qi Wu, Rong Deng, Dan-Dan Li, Jun Tang, Wen-Dan Chen, Jing-Hong Chen, Jiao Ji, Lin Jiao, Shan Jiang, Fen Yang, Gong-Kan Feng, Ravichandran Senthilkumar, Fei Yue, Hai-Liang Zhang, Rui-Yan Wu, Yan Yu, Xue-Lian Xu, Jia Mai, Zhi-Ling Li, Xiao-Dan Peng, Yun Huang, Xiang Huang, Ning-Fang Ma, Qian Tao, Yi-Xin Zeng, Xiao-Feng Zhu

**Affiliations:** 1 State Key Laboratory of Oncology in South China, Collaborative Innovation Center for Cancer Medicine, Cancer Center, Sun Yat-sen University, 510060 Guangzhou, China; 2 The 3rd Affiliated Hospital, Sun Yat-sen University, 510620 Guangzhou, China; 3 Department of Hematology, The Second Affiliated Hospital, Guangzhou Medical University, 510260 Guangzhou, China; 4 Department of Oncology, The First Affiliated Hospital, Chongqing Medical University, 400016 Chongqing, China; 5 Department of Biochemistry and Molecular Biology, Nanjing Medical University, 211166 Nanjing, China; 6 Institute of Biosciences and Technology, Texas A&M University Health Science Center, Houston, TX 77030 USA; 7 The School of Medicine, Jinan University, 510632 Guangzhou, China; 8 Key Laboratory of Protein Modification and Degradation, School of Basic Medical Sciences, Affiliated Cancer Hospital and Institute of Guangzhou Medical University, 511436 Guangzhou, China; 9 Sir YK Pao Cancer Centre, Department of Clinical Oncology, Prince of Wales Hospital, The Chinese University of Hong Kong, Hong Kong SAR, China

## Abstract

Autophagy is a degradative pathway that delivers cellular components to the lysosome for degradation. The role of autophagy in cell differentiation is poorly understood. Here we show that CaMKII can directly phosphorylate Beclin 1 at Ser90 to promote K63-linked ubiquitination of Beclin 1 and activation of autophagy. Meanwhile, CaMKII can also promote K63-linked ubiquitination of inhibitor of differentiation 1/2 (Id-1/2) by catalyzing phosphorylation of Id proteins and recruiting TRAF-6. Ubiquitinated Id-1/Id-2 can then bind to p62 and be transported to autolysosomes for degradation. Id degradation promotes the differentiation of neuroblastoma cells and reduces the proportion of stem-like cells. Our study proposes a mechanism by which autophagic degradation of Id proteins can regulate cell differentiation. This suggests that targeting of CaMKII and the regulation of autophagic degradation of Id may be an effective therapeutic strategy to induce cell differentiation in neuroblastoma.

## Abstract (web summary)

Neuroblastoma cell differentiation is regulated by Id proteins. Here, the authors show that CaMKII-mediated phosphorylation of Beclin 1 can activate K63-linked ubiquitination and autophagic degradation of Id proteins uncovering a role for autophagy in cell differentiation.

## Introduction

Macroautophagy (hereafter referred to as autophagy) is a biological process in which the massive degradation of cytoplasmic macromolecules and organelles occurs in membrane vesicles under metabolic stress, such as hunger and energy deficiency^1^. The products of degradation, including amino acids, nucleotides, and free fatty acids, can be introduced into the energy cycle and re-used by cells to maintain normal metabolism and cell survival. Autophagy can also be used as a defense mechanism to remove damaged or excess metabolites in the cytoplasm, alleviate the accumulation of abnormal proteins and organelles, and protect damaged cells^2^. Autophagy is closely associated with a variety of human diseases, such as malignancies, neurodegenerative diseases, myopathies, and infectious diseases^3–7^.

To date, more than 30 yeast-specific genes implicated in autophagy have been identified; these genes are known as the ATG (AuTophaGy-related) genes. As research has progressed, numerous yeast autophagy-related gene homologs have been identified in mammals, suggesting that autophagy is an evolutionarily conserved process^8^. The occurrence of autophagy is regulated by the ATGs, which in turn are modulated by other intracellular signaling pathways^9^. Recent studies have demonstrated that the regulation of autophagy initiation is mainly mediated by two key complexes: ULK1 and Beclin 1^10^. Membrane elongation and autophagosome completion requires two ubiquitin-like conjugation pathways, the ATG5–ATG12 and LC3–PE conjugate^11^. In the process of autophagy, autophagosome formation is the most complex stage. Beclin 1 and its binding proteins are critical in this stage. The expression and activity of the Beclin 1 complex are closely related to the occurrence of autophagy^12–16^.

As the first autophagy-related gene found in mammals, Beclin 1 is the mammalian homolog of yeast *Atg6*, which contains four major structural domains^17^. Beclin 1 binds to hVps34 (PI3K III) to form complexes that can promote the recruitment of other autophagy-related proteins such as the positive factor Atg14L, UVRAG which can promote autophagy, and the negative factor Bcl-2, Rubicon which can inhibit autophagy. Beclin 1 is like a scaffold protein that can be modified during the whole autophagy process. Under conditions of hunger, JNK1 can directly phosphorylate Bcl-2 to release free Beclin 1 from the Bcl-2/Beclin 1 complex, thus regulating autophagy^18^. Many other kinases, such as AMPK, Akt, EGFR, and MK2 could regulate Beclin 1-Vps34 complex through phosphorylating Beclin 1. Besides phosphorylation, other modification like ubiquitylation and acetylation of Beclin 1 also affect the formation of Beclin 1 complex. In addition to the role of Beclin 1 in autophagy, it also functions as a tumor suppressor in mammalian cells^12^. Beclin 1 is monoallelically deleted in a high percentage of human breast, ovarian, and prostate cancers, and its expression suppresses the tumorigenicity of human cancer cell lines^12^. Wirawan et al. demonstrated their significant findings that caspases can cleave Beclin 1, thereby destroying its pro-autophagic activity^19^. Moreover, the C-terminal fragment of Beclin 1 that results from this cleavage acquires a new function and can amplify mitochondrion-mediated apoptosis^20^.

Calcium/calmodulin-dependent protein kinase II (CaMKII) is a serine/threonine protein kinase that is a member of the CaMK family and functions as an important calcium signaling molecule^21^. Many calcium-mobilizing agents have been reported to activate Ca^2+^/CaM and downstream calcium signaling pathways, including CaMKII. For example, ionomycin is a commonly used calcium ionophore^22^. In addition, EB1089 (seocalcitol) is a newly synthesized vitamin D analog that can release calcium ions from inside the endoplasmic reticulum into the cell cytoplasm, resulting in an increase in the intracellular calcium concentration^23^; this compound has undergone clinical phase I and II trials and was found to be well tolerated.

In this study, we examine the effects of CaMKII on Beclin 1-induced autophagy. We find that CaMKII could directly phosphorylate Beclin 1 at Ser90 and activate autophagy. CaMKII cause the K63-linked ubiquitination of Id-1/2 mediated by TRAF-6, which is involved in the phosphorylation of Id Ser36. Ubiquitinated Id-1/2 then bound to p62, and the complex is delivered to autolysosomes for degradation. Furthermore, Id degradation promotes the differentiation of neuroblastoma cells and reduce the proportion of stem-like cells. This study sheds light on the relevance between autophagy and differentiation under CaMKII activation and provides insight into the mechanism and roles of autophagy in solid tumor cell differentiation.

## Results

### CaMKII affects the phosphorylation of Beclin 1 at Ser90

**Fig. 1:**
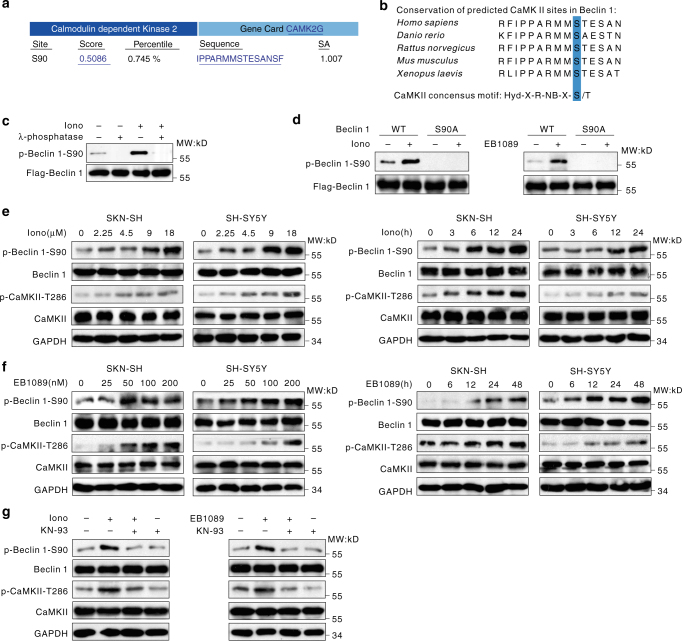
CaMKII activity affects the phosphorylation of Beclin 1 at Ser90. **a** Based on an analysis of the Beclin 1 amino acid sequence, Ser90 of Beclin 1 is strongly predicted to be a CaMKII phosphorylation site. **b** The predicted CaMKII phosphorylation sites in Beclin 1 are highly conserved during evolution. Putative CaMKII phosphorylation sites are indicated with a blue background. **c** The specificity of the anti-phospho-Ser90 Beclin 1 antibody. HEK293T cells were transiently transfected with plasmids encoding Flag-Beclin 1-WT, and the cells were incubated for 1 h at 30 °C in the absence or presence of ionomycin. The lysates of cells were incubated with or without λ-phosphatase. Flag-tagged proteins were immunoprecipitated and analyzed. **d** Ca (2+) mobilizing agents regulate Beclin 1 Ser90 phosphorylation. HEK293T cells were transiently transfected with the indicated plasmids for 48 h and then lysed, and Beclin 1 was precipitated using an anti-Flag antibody. The precipitates were analyzed by western blotting using an anti-phospho-Beclin 1-S90 antibody. **e** Ionomycin-induced phosphorylation of Beclin 1 on Ser90 occurs via a CaMKII-dependent pathway in a time-dependent and concentration-dependent manner. Both cell lines were treated with 6 μM ionomycin for the indicated periods or with various concentrations of ionomycin for 24 h. The cell extracts were analyzed by western blotting. **f** The EB1089-induced phosphorylation of Beclin 1 on Ser90 occurs via a CaMKII- dependent pathway in a time-dependent and concentration-dependent manner. Both cell lines were treated with 100 nM EB1089 for the indicated periods or various concentrations of EB1089 for 24 h. The cell extracts were analyzed by western blotting. **g** The inhibition of the phosphorylation of Beclin 1 by a CaMKII inhibitor. SK-N-SH cells were untreated or treated with 6 μM ionomycin, 100 nM EB1089, or 10 μM KN-93 for 24 h. The cell extracts were analyzed by western blotting

Using the Scansite software^24^, we predicted that Beclin 1 may be phosphorylated at residue Ser90 by CaMKII (Fig. 1a). We analyzed the motif containing Ser90 in Beclin 1 from residue 81 to residue 95 (RFIPPARMMS★TESAN), and found that this sequence is highly conserved in evolution and follows the sequence pattern of CaMKII phosphorylation substrates^25^ (Fig. 1b). This finding suggests that CaMKII has the potential ability to phosphorylate Beclin 1 at Ser90.

To test this hypothesis, we performed a mass spectrometry analysis after immunoprecipitation of Beclin 1 from 293T cells. The result showed that Beclin 1 Ser90 was phosphorylated when co-transfected with CaMKII (Supplementary Fig. 1c). Furthermore, we prepared an antibody that specifically recognizes phosphorylated Ser90 of Beclin 1 (Supplementary Fig. 1a). Immunoreactive proteins were detected using this phospho-specific Beclin 1 antibody in immunoprecipitates isolated using an anti-Flag antibody from SK-N-SH cells that express wild-type Flag-Beclin 1 (WT) plasmid (Fig. 1c). However, these signals were not observed after treating with λ-phosphatase (PPase), a phosphatase capable of removing phosphates from serine, threonine, and tyrosine residues (Fig. 1c). In contrast, this PPase-sensitive modification could be restored to pre-treatment levels by rescuing with phosphatase inhibitors. Meanwhile, after treatment with ionomycin, enhanced phosphorylation of Beclin 1 at Ser90 was detected in human embryonic kidney (HEK) 293 cells transfected with the Flag-Beclin 1-WT plasmid. However, in cells that were transfected with the Flag-Beclin 1-S90A plasmid, no phosphorylation of Beclin 1 at Ser90 was detected either with or without ionomycin treatment (Fig. 1d). These results clearly exhibited that Beclin 1 was phosphorylated at Ser90 and the antibody for phosphorylated Beclin 1 at Ser90 was specific.

Ionomycin, EB1089, and Vitamin D3, three calcium-mobilizing agents with different mechanisms of action, were used to activate CaMKII in the cells. In four different neuroblastoma cell lines, CaMKII was effectively activated and the phosphorylation of Beclin 1 at Ser90 was also significantly increased (Fig. 1e, f, Supplementary Fig. 1b, 6d). This result demonstrated that the activation of CaMKII could boost Beclin 1 phosphorylation at Ser90. Meanwhile, there was no significant change in Beclin 1 mRNA expression with respect to control as detected by real-time RT-PCR following the treatment (Supplementary Fig. 1d).

**Fig. 2:**
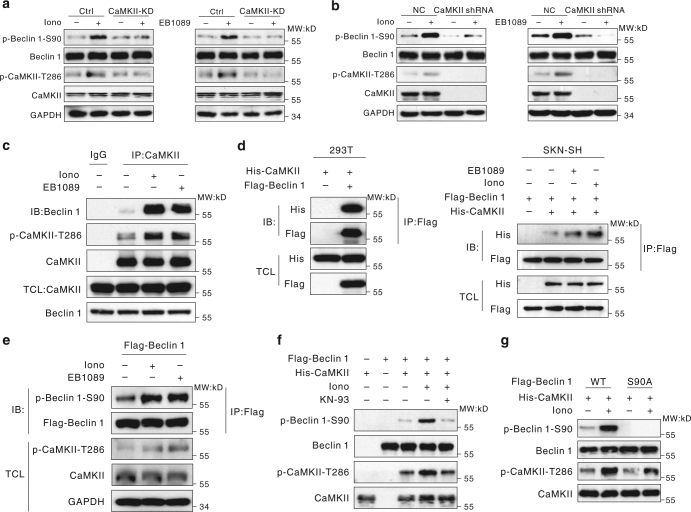
CaMKII directly phosphorylates Beclin 1 at Ser90. **a** The phosphorylation of Beclin 1-S90 requires activated CaMKII. SK-N-SH cells were transiently transfected with a control vector or plasmids encoding His-CaMKII-KD for 48 h. The whole-cell extracts were subjected to western blotting using the indicated antibodies. **b** CaMKII is required for phosphorylation of Beclin 1 on S90. HEK293T cells were transiently transfected with negative control or CaMKII shRNA, and then untreated or treated with 6 μM ionomycin or 100 nM EB1089 for 72 h. The cells were lysed and the indicated proteins were analyzed by western blot. **c** Increased association between CaMKII and Beclin 1 in cells treated with ionomycin or EB1089. HEK293T cells were treated with 6 μM ionomycin or 100 nM EB1089 for 24 h, lysed, and then incubated with anti-CaMKII or control IgG antibodies prior to western blotting. The resulting immunoprecipitates (IP) were subjected to western blot analysis. TCL, the whole-cell lysate. **d** The association between His-tagged CaMKII and Flag-tagged Beclin 1. HEK293T cells or SK-N-SH cells were transiently co-transfected with plasmids encoding Flag-Beclin 1-WT and His-CaMKII-WT, lysed, and incubated with an anti-Flag antibody prior to western blot. The resulting immunoprecipitates were subjected to western blot analysis. TCL, the whole-cell lysate. **e** Phosphorylation of Beclin 1 by CaMKII upon ionomycin or EB1089 treatment. HEK293T cells were transiently transfected with plasmids encoding Flag-Beclin 1-WT, and the cells were incubated in 6 μM ionomycin or 100 nM EB1089. Flag-tagged proteins were precipitated and analyzed by western blot using an anti-phospho-S90-Beclin 1 and other indicated antibodies. **f** The phosphorylation-dependent activity of CaMKII on Beclin 1. HEK293T cells transfected with His-CaMKII-WT were untreated or treated with 6 μM ionomycin or 10 μM KN-93 for 24 h, then lysed and incubated with an anti-His antibody. Purified Beclin 1 fusion proteins were incubated with the immunoprecipitate, and in vitro kinase assays were performed. **g** In vitro phosphorylation of Beclin 1. HEK293T cells transfected with His-CaMKII-WT were treated with 6 μM ionomycin, then lysed and incubated with an anti-His antibody. Purified Beclin 1-WT and Beclin 1-S90A fusion proteins were incubated with the immunoprecipitate, and in vitro kinase assays were performed

Lastly, KN-93, a specific CaMKII inhibitor^26^, was used to examine the effect of CaMKII on the phosphorylation of Beclin 1. In SK-N-SH cells that were pre-incubated with KN-93, the addition of ionomycin and EB1089 could not activate CaMKII, and the level of Beclin 1 Ser90 phosphorylation was not altered (Fig. 1g). To further validate this finding, we constructed a His-CaMKII-KD (kinase-inactive) plasmid; this CaMKII mutant could not enhance the phosphorylation of Beclin 1 at Ser90 (Fig. 2a). Most important of all, knockdown of CaMKII could impair the effect of ionomycin or EB1089 on phosphorylation of Beciin 1 at Ser90 (Fig. 2b, Supplementary Fig. 6a). Taken together, these results clearly confirmed that CaMKII activation was necessary for the increased phosphorylation of Beclin 1 at Ser90.

### CaMKII directly phosphorylates Beclin 1 at Ser90

We next evaluated whether CaMKII binds to Beclin 1 in tumor cells. The immunoprecipitation experiments showed that endogenous Beclin 1 bound with CaMKII (Fig. 2c). In both 293T and SK-N-SH cells that were transiently co-transfected with WT His-CaMKII and Flag-Beclin 1 plasmids, similar results were obtained (Fig. 2d). To further verify the interaction, an in vitro binding assay was made. As expected, purified His-CaMKII was pulled down by Flag-Beclin 1, which indicated a strong direct interaction between these two proteins (Supplementary Fig. 2e). These findings demonstrated that either endogenous or exogenous CaMKII can bind to Beclin 1.

Furthermore, the phosphorylation on Beclin 1 Ser90 was intensified in HEK293T cells treated with ionomycin or EB1089 (Fig. 2e), confirming the hypothesis that CaMKII regulates Beclin 1 Ser90 phosphorylation.

Finally, the in vitro kinase assays were performed. HEK293T cells were treated with ionomycin, CaMKII was immunoprecipitated, and the resultant immunoprecipitation complexes were mixed with purified WT Beclin 1 fusion protein. CaMKII could direct phosphorylate Beclin 1 at Ser90 (Fig. 2f). The addition of the CaMKII inhibitor KN-93 attenuated the CaMKII-induced phosphorylation of Beclin 1 at Ser90. We also purified a mutant Beclin 1-S90A fusion protein and found that CaMKII failed to phosphorylate the mutant Beclin 1 (Fig. 2g). Importantly, Western blot results showed that the Beclin 1 fusion protein was efficiently phosphorylated when incubated with recombinant active CaMKII; however, the Beclin 1 fusion protein containing the S90A mutant was scarcely phosphorylated under the same conditions (Supplementary Fig. 2f). These findings suggested that CaMKII can directly phosphorylate Beclin 1 at Ser90.

### CaMKII induces autophagy through phosphorylating Beclin 1

**Fig. 3:**
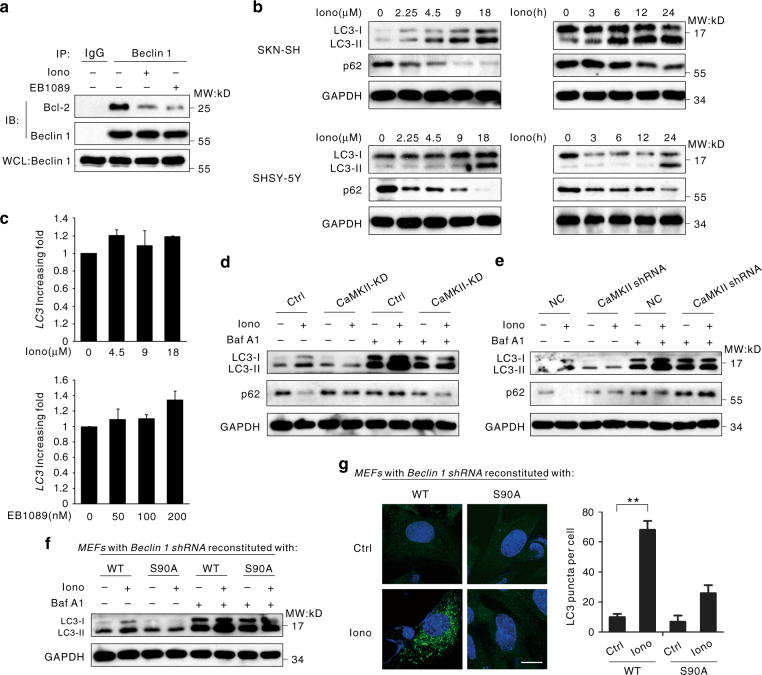
CaMKII induces autophagy through phosphorylation of Beclin 1 at Ser90 and the subsequent ubiquitination at Lys117. **a** Dissociation of the Bcl-2-Beclin 1 Complex. SK-N-SH cells treated with 6 μM ionomycin or 100 nM EB1089 were lysed and incubated with anti-Beclin 1 or normal mouse IgG antibodies prior to western blot analysis. The resulting immunoprecipitates were subjected to western blot analysis using the indicated antibodies. TCL, whole-cell lysate. **b** Autophagy induced by ionomycin in neuroblastoma cells. Both cell lines were treated with 6 μM ionomycin for the indicated periods or various concentrations of ionomycin for 24 h. The total cell lysates were analyzed for LC3 lipidation by immunoblotting. **c** LC3 mRNA expression was unaffected in cells treated with ionomycin or EB1089. The SK-N-SH cells were untreated or were treated with ionomycin or EB1089 of indicated concentration for 24 h. The expression level of LC3 mRNA was detected by real-time RT-PCR. The error bars represent the standard deviations (SD) calculated from three parallel experiments. **d** LC3 lipidation and p62 degradation in CaMKII-KD-expressing MEFs cells or control cells (Ctrl). MEFs cells transfected with a control vector or plasmids encoding His-CaMKII-KD were treated with 6 μM ionomycin for 24 h, then incubated for 2 h in the presence or absence of 100 nM Baf A1. The whole-cell extracts were subjected to western blot using the indicated antibodies. **e** LC3 lipidation and p62 degradation in CaMKII shRNA MEFs cells or negative control cells (NC). MEFs cells transfected with negative control or CaMKII shRNA were treated with 6 μM ionomycin for 24 h, then incubated for 2 h in the presence or absence of 100 nM Baf A1. The whole-cell extracts were subjected to western blot using the indicated antibodies. **f** LC3 lipidation in Beclin 1-deficient MEFs expressing WT Beclin 1 or S90A-Beclin 1. The cells were incubated in 6 μM ionomycin for 24 h, and total cell lysates were analyzed for LC3 lipidation by means of immunoblotting. **g** Autophagosome formation in MEFs deficient for Beclin 1. The cells were incubated in 6 μM ionomycin for 24 h and then fixed. LC3-positive puncta were shown as the means ± SEM of five random areas. **P < 0.01, Student’s *t*-test. Scale bar, 10 μm

Previous studies have shown that Beclin 1 interacts with Bcl-2 family members via amino acids 88–140, the BH3 domain^27^. If the two proteins disassociate with each other, Bcl-2 will lose its inhibitory function on Beclin 1-mediated autophagy^28^. We noted that Ser90 of Beclin 1, which was phosphorylated by CaMKII, was exactly located within the Beclin 1-binding sequence in Bcl-2. Treatment of SK-N-SH cells with ionomycin and EB1089 decreased the association between Bcl-2 and Beclin 1 (Fig. 3a). To further verify the interaction between Beclin 1 and other Beclin 1-binding proteins, Flag-Beclin 1-WT and its mutants were overexpressed in HEK293T cells. As shown in Supplementary Fig. 2a, ionomycin largely diminished the binding between Beclin 1 and Bcl-2, which was restored in cells overexpressing Flag-Beclin 1-S90A. As expected, ionomycin treatment also enhanced the interaction between Beclin 1 and Vps34 (Supplementary Fig. 2a). However, stronger interaction between Bcl-2 and Beclin 1 was observed upon KN-93 treatment, which was impaired after Beclin 1-S90D transfection (Supplementary Fig. 2b). Accordingly, the association between Vps34 and Beclin 1 was repaired in cells overexpressing Beclin 1-S90D (Supplementary Fig. 2b). These findings suggest that Beclin 1 disassociates from Bcl-2 and may release the Bcl-2-mediated inhibition of autophagy. We next exposed four neuroblastoma cell lines to ionomycin or Vitamin D3 and observed that both this two agents increased the expression of LC3-II or LC3-II punctate foci (Fig. 3b, Supplementary Figs. 2c, d, 6d)^29^. As detected by Q-PCR, there was no statistically significant difference of LC3 transcription between ionomycin/EB1089 treated cells and control cells (Fig. 3c). Taken together, the Ca^2+^ mobilization agents could induce autophagy by affecting the formation of Beclin 1 complex.

To examine whether ionomycin-induced autophagy is associated with CaMKII activation, we transfected the His-CaMKII-KD plasmid in mouse embryonic fibroblast (MEF) cells and found that ionomycin-induced autophagy was significantly decreased (Fig. 3d). Similarly, CaMKII knockdown had a significant effect on ionomycin-induced LC3-II enhancement (Fig. 3e, Supplementary Fig. 6a). However, in MEF cells transfected with His-CaMKII-CA plasmid, the expression of LC3-II was enhanced comparing with the cells transfected with Vector plasmid (Supplementary Fig. 3b). Next, we used the autolysosome fusion inhibitor bafilomycin A1 to exclude the effect of lysosomal activity on autophagy and monitor a real change in autophagic flux in the cells^30^. After treatment with bafilomycin A1, the expression of LC3-II was further increased, suggesting that ionomycin-induced autophagy requires CaMKII activation (Fig. 3d, e, Supplementary Fig. 3b).

It has been widely reported that Beclin 1 could be phosphorylated by different kinases in different conditions. Wei et al.^31^ have reported that MAPKAPK2 (MK2) and MAPKAPK3 (MK3), positively regulate starvation-induced autophagy by phosphorylating Beclin 1 at serine 90. The results of Supplementary Figs. 3d–f, 4a–d showed that knockdown/inhibition of MK2/3/5 or p38 failed to impact the high level of Beclin 1 phosphorylation and autophagy in normal or ionomycin-induced condition. Which indicated that MKs did not function during the ionomycin-induced autophagy. Also it has been reported that the Ca^2+^/CaMKKbeta/AMPK/mTOR pathway activates autophagy in response to ionomycin^32^, AMPK activation or mTOR inhibition in the current study was marginal (Supplementary Fig. 3c). This discrepancy may be due to the different cell lines and different conditions utilized in these studies.

Next, MEF cells with stable silencing of Beclin 1 were reconstituted with the Flag-Beclin 1-WT or Flag-Beclin 1-S90A plasmids, respectively. The results showed that ionomycin could induce autophagy in WT Beclin 1 cells. In contrast, autophagy was significantly suppressed in the cells with mutant Beclin 1-S90A (Fig. 3f). Furthermore, autophagy flux blockade by CaMKII inactivation could be reversed by Beclin 1-S90D (Supplementary Fig. 3a).

To further verify this result, the cells stably expressing the pBabe-pEYFP-LC3 plasmid were constructed to observe formation of LC3-II punctate foci. Consistent with our above results, the Beclin 1-S90A mutation greatly reduced the formation of autophagic punctate foci (Fig. 3g). This result demonstrated that ionomycin-induced autophagy required the phosphorylation of Beclin 1 at Ser90.

Shi and Kehrl^33^ reported that TRAF-6 interacts with and mediates K63-linked ubiquitination of Beclin 1 Lys117, and this activity is important for Beclin 1 dependent autophagy. In our study, Beclin 1 is also a substrate of TRAF-6 (Supplementary Fig. 5a). Therefore, we tried to find out whether phosphorylation of Beclin 1 at Ser90 by CaMKII affects its ubiquitination. The results revealed that Ser90 phosphorylation could increase the TRAF-6-mediated K63-ubiquitination on Beclin 1 (Supplementary Figs. 5a–c, 7a). Interestingly, the K117R mutant Beclin 1 could reverse the autophagy level stimulated by ionomycin with a reduced expressing of LC3-II or the loss of EYFP-LC3-II puncta (Supplementary Fig. 7b–d). It suggests that phosphorylation and subsequentially K63-ubiquitination of Beclin 1 both contribute to autophagy induction by ionomycin or EB1089.

### Id-1/2 are degraded via autophagy

Although the pathogenesis is not completely clear, it has been reported that many genetic susceptibility factors are associated with neuroblastoma such as the somatic mutation of *ALK* and the amplification mutation of *N-myc*. In this study, the neuroblatoma cells were exposed to ionomycin and EB1089, and the protein levels of ALK and N-myc have no obvious change (Supplementary Fig. 8a).

**Fig. 4:**
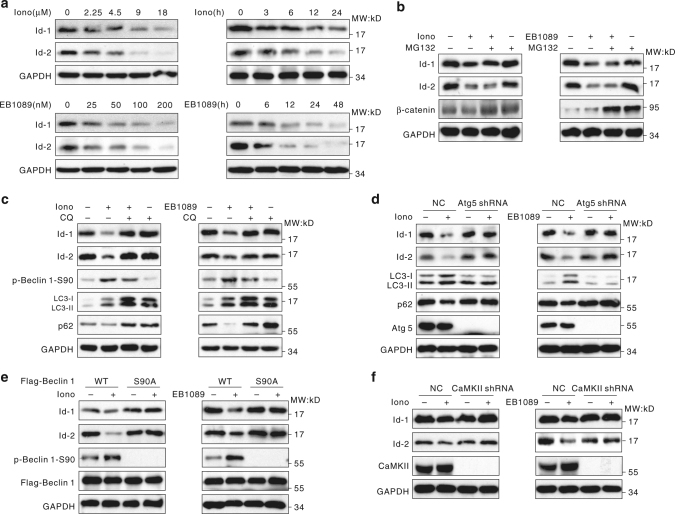
Autophagy induced by CaMKII promotes degradation of inhibitor of differentiation proteins. **a** The degradation of Id-1 and Id-2 after ionomycin and EB1089 treatment. SK-N-SH cells were treated with 6 μM ionomycin or 100 nM EB1089 for the indicated periods or various concentrations of ionomycin or EB1089 for 24 h. The whole-cell lysates were analyzed by immunoblotting. **b** The ionomycin- and EB1089-induced degradation of Id-1 and Id-2 does not occur via the proteasome pathway. SK-N-SH cells were untreated or treated with 6 μM ionomycin or 100 nM EB1089 for 24 h and then incubated for 4 h in the presence or absence of 10 μM MG132. The total cell extracts were subjected to western blot using the indicated antibodies. **c** Autophagy is involved in the ionomycin-/EB1089-induced degradation of Id-1 and Id-2. SK-N-SH cells were untreated or treated with 6 μM ionomycin or 100 nM EB1089 for 24 h, then incubated for 2 h in the presence or absence of 10 μM chloroquine (CQ). The whole-cell extracts were subjected to western blotting using the indicated antibodies. **d** Id-1/2 proteins are degraded via the autophagy pathway. SK-N-SH cells were transfected for 24 h with a negative control (NC) or Atg5 shRNA and then incubated for 24 h with 6 μM ionomycin or 100 nM EB1089. The cell lysates were then analyzed by western blotting. **e** The phosphorylation of Beclin 1 Ser90 is essential for the ionomycin-/EB1089-induced degradation of Id-1 and Id-2. HEK293T cells were transiently transfected with the indicated plasmids for 24 h and then treated with 6 μM ionomycin or 100 nM EB1089 for 24 h. The whole-cell extracts were subjected to western blotting using the indicated antibodies. **f** CaMKII is required for the degradation of Id-1 and Id-2 after ionomycin and EB1089 treatment. HEK293T cells were transiently transfected with negative control or CaMKII shRNA for 24 h and then treated with 6 μM ionomycin or 100 nM EB1089. The whole-cell extracts were subjected to western blotting using the indicated antibodies

As a kind of poorly differentiated solid tumors occurring during infancy, neuroblastoma shows the potential of developing sympathetic neuroblasts. Also neuroblastoma cell lines can be induced to differentiate in vitro by several agents, including retinoic acid (RA), which is frequently applied in clinics^34, 35^. Jogi et al.^36^ reported that the three Id (the inhibitor of differentiation) proteins expressed in neuroblastoma cells (Id-1, Id-2, and Id-3) were downregulated during induced differentiation, indicating that Id proteins helped to keep the tumor cells in an undifferentiated state. Hence Ids were taken into account as a target for treatment of neuroblastoma by inducing cell differentiation artificially. As shown in Fig. 4a, with ionomycin and EB1089 treatment, the protein levels of Id-1 and Id-2 were significantly reduced. While Id-1 and Id-2 mRNA levels did not exhibit significant changes in the treated cells (Supplementary Fig. 8c). This finding suggested that ionomycin and EB1089 might regulate Id-1 and Id-2 protein levels by affecting their degradation.

To evaluate whether the proteasome degradation pathway is involved in Id-1/2 degradation, the proteasome inhibitor MG132 was utilized. MG132 failed to reverse the degradation of the Id-1 and Id-2 proteins in response to ionomycin and EB1089 (Fig. 4b), as same as another proteasome inhibitor bortezomib (Supplementary Fig. 8b), thereby excluding the possibility that these two proteins were degraded via the proteasome pathway.

Next, we speculated that the autophagy-lysosome degradation pathway might be involved in Id-1/2 degradation. The lysosome inhibitor chloroquine (CQ) was used to block autophagic degradation. As shown in Fig. 4c, in contrast to the results with MG132 treatment, CQ significantly reversed the ionomycin/EB1089-induced Id-1 and Id-2 protein degradation. Similarly, Id-1 and Id-2 were not degraded in cells lacking Atg5 (Fig. 4d, Supplementary Fig. 6b), proving that Id-1 and Id-2 proteins were degraded through the autophagy-lysosome pathway.

Because the Id-1 and Id-2 proteins were degraded through the autophagy-lysosome pathway, it was important to determine whether this degradation is related to CaMKII and the phosphorylation of Beclin 1 at Ser90. When Beclin 1 Ser90 was mutated to alanine, ionomycin/EB1089 exposure failed to cause Id-1 and Id-2 degradation (Fig. 4e). In addition, CaMKII silencing could block the downregulation of Id-1/2 induced by ionomycin or EB1089 (Fig. 4f, Supplementary Fig. 6a). These findings indicated that Id-1 and Id-2 proteins were degradated via autophagy and this process required the phosphorylation of Beclin 1 at Ser90.

### K63-linked ubiquitylation facilitates Id-1/2 degradation

**Fig. 5:**
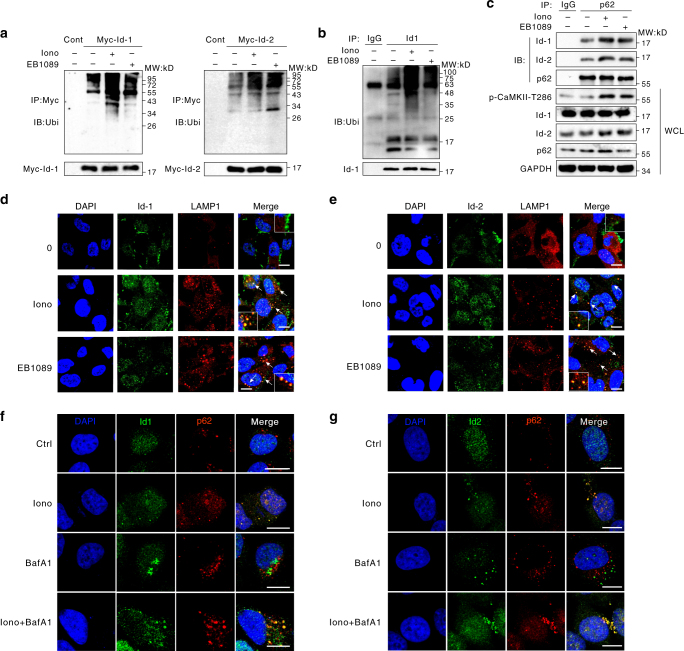
The Lys-63-linked ubiquitylation of inhibitor of differentiation proteins are essential to their autophagic degradation. **a** Ionomycin and EB1089 enhanced Id-1/2 ubiquitination. HEK293T cells were transiently transfected with the indicated plasmids, treated with 6 μM ionomycin or 100 nM EB1089 for 24 h, and then incubated in 100 nM Baf A1 for 4 h. Immunoprecipitated Myc-Id-1/2 was subjected to anti-ubiquitin western blotting. **b** Ionomycin and EB1089 enhanced endogenous Id-1 ubiquitination. SK-N-SH cells were treated with 6 μM ionomycin or 100 nM EB1089 for 24 h, and then incubated in 100 nM Baf A1 for 4 h. Immunoprecipitated Id-1 was subjected to anti-ubiquitin western blotting. **c** Id-1 and Id-2 interact with p62. HEK293T cells treated with 6 μM ionomycin or 100 nM EB1089 for 24 h and 100 nM Baf A1 for 6 h before collect. The protein extracts were incubated with anti-p62 or normal mouse IgG antibodies prior to western blot. The resulting immunoprecipitates (IP) were subjected to western blot analysis using the indicated antibodies. WCL, whole-cell lysate. **d** Id-1 was enclosed in LAMP1-positive autolysosomes. MEFs cells were transiently transfected with Myc-Id-1 and then incubated with 6 μM ionomycin or 100 nM EB1089 for 24 h. Confocal images of transfected cells representing the co-localization of Id-1 with the lysosome marker LAMP1. The merged images and relative insets reveal the co-localization. Scale bar, 10 μm. **e** Id-2 was enclosed in LAMP1-positive autolysosomes. MEFs cells were transiently transfected with Myc-Id-2, then incubated with 6 μM ionomycin or 100 nM EB1089 for 24 h. Confocal images of transfected cells representing the co-localization of Id-2 with the lysosome marker LAMP1. The merged images and relative insets reveal the co-localization. Scale bar, 10 μm. **f**, **g** Id-1 and Id-2 was recruited by p62. MEFs cells were incubated with 6 μM ionomycin for 24 h and Baf A1 for 2 h. Confocal images of cells representing the co-localization of Id-1/2 with p62. The yellow puncta of the merged images reveal the co-localization. Scale bar, 10 μm

In mammalian cells, ubiquitination is the degradation signal for proteins to be degraded via either the proteasome pathway or the autophagy pathway. To examine the ubiquitination of the Id proteins, HEK293T cells were transfected with Myc-Id-1 or Myc-Id-2 plasmids. Following the treatment with ionomycin or EB1089, the results of immunoprecipitation assay revealed that Id-1 and Id-2 ubiquitination was enhanced significantly (Fig. 5a). And in SK-N-SH cells, Id-1 ubiquitination was enhanced by the two CaMKII activators, even without overexpressing (Fig. 5b).

Meanwhile, as shown in Fig. 5c, following treatment with ionomycin and EB1089, the association between Id-1/2 and p62 was significantly increased. To study the subcellular localization of Id-1 and Id-2, confocal microscopy analyses were performed using an organelle-specific protein marker^37^. Under steady-state conditions, Id-1 and Id-2 were diffusely localized throughout the cytosol and did not overlap with LAMP1 (the lysosome marker) or p62, but Id-1 and Id-2 could rapidly associate with p62 and were recruited to the lysosome after ionomycin or EB1089 treatment (Fig. 5d–g). These results suggest that p62 could recognize and bind to ubiquitinated Id-1 and Id-2 and that the Id-p62 complex was then transported to autophagosomes for degradation.

**Fig. 6:**
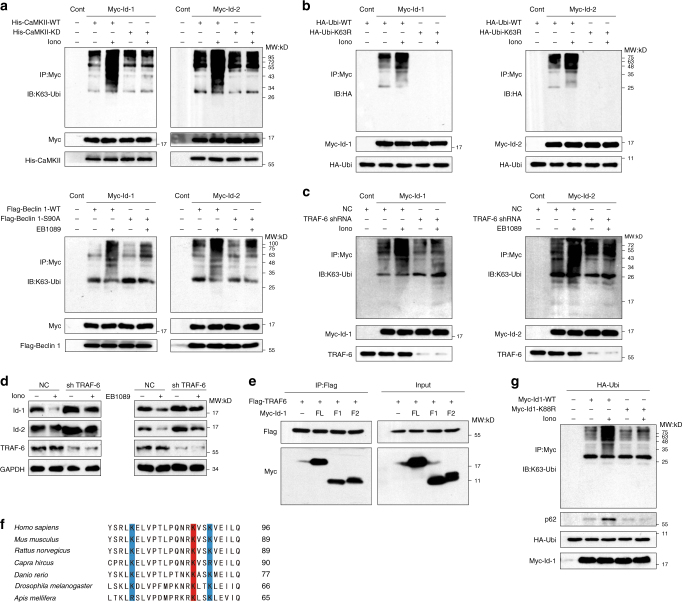
TRAF-6 induced the K63-linked ubiquitination of Id-1 at the K88 residue. **a** CaMKII and phosphorylation of Beclin 1 Ser90 are required for K63 ubiquitination of Ids. HEK293T cells were transiently co-transfected with the indicated plasmids, treated with 6 μM ionomycin for 24 h, and then incubated with 100 nM Baf A1 for 4 h. The immunoprecipitated Myc-Ids was subjected to anti-K63-ubiquitin western blot. **b** Id-1 and Id-2 undergoes K63 ubiquitination. HEK293T cells were transiently transfected with HA-Ubi or HA-Ubi-K63R plasmids, treated with 6 μM ionomycin or 100 nM EB1089 for 24 h, and then incubated with 100 nM Baf A1 for 4 h. The immunoprecipitated Myc-Id-1/2 was subjected to anti-HA western blot. **c** TRAF-6 is required for the K63-ubiquitination of Id-1/2. HEK293T cells were transfected with the indicated plasmids for 24 h, incubated with 6 μM ionomycin or 100 nM EB1089 for 24 h, and then treated with Baf A1 for 4 h. The immunoprecipitated Myc-Id-1/2 was subjected to anti-HA western blot. **d** TRAF-6 is required for the degradation of Id-1/2. HEK293T cells were transfected for 24 h with the negative control (NC) or TRAF-6 shRNA and then incubated for 24 h with 6 μM ionomycin or 100 nM EB1089. The cell lysates were then analyzed by western blot. **e** The bHLH domain of Id-1 was required for its interaction with TRAF-6. HEK293T cells were transiently transfected with the indicated plasmids for 24 h and then treated with 6 μM ionomycin for 24 h and 100 nM Baf A1 for 2 h before lysis. The whole-cell extracts were subjected to western blotting using the indicated antibodies after immunoprecipitation. **f** The lysine88 residues in the bHLH domain of Id-1 is more conserved during evolution (indicated with a red background and the other two with a blue background). **g** The K88 residue of Id-1 was necessary to the K63-linked ubiquitination of Id-1 induced by ionomycin. SK-N-SH cells were transiently co-transfected with plasmids encoding Myc-Id-1-WT or K88R and plasmid with HA-Ubiquitin, then treated with 6 μM ionomycin for 24 h, and with Baf A1 for 4 h. The cell lysates were then analyzed by western blot after the immunoprecipitation

Polyubiquitination via K29 or K48 suggests that the substrate proteins will be destined for proteasome-mediated degradation. Mono-ubiquitination or polyubiquitination via K63 suggests that substrate proteins will be recognized by specific regulatory factors to trigger the corresponding signaling pathways^38^. In addition, the p62 protein has a strong affinity for the K63-linked polyubiquitin chain^39–41^, so we speculated that ionomycin and EB1089 treatment would induce the K63-linked polyubiquitination of Id-1 and Id-2. As shown in Fig. 6a, Id-1 showed basal levels of K63-linked polyubiquitination, which was enhanced after treatment with ionomycin. Moreover, when the function of CaMKII was compromised or Beclin 1 was mutant, K63-linked polyubiquitination of Id-1 significantly decreased (Fig. 6a). To further clarify whether ubiquitination occurred through K63, the ubiquitin K63R mutant was introduced to the cells. As shown in Fig. 6b, ionomycin or EB1089 treatment failed to promote ubiquitination of Id-1 and Id-2 in cells overexpressing ubiquitin-K63R. These findings indicated that the degradation of Ids was mediated by the K63-linked ubiquitination, and the activation of CaMKII was required.

To search for the E3 ubiquitin ligase that plays a key role in this process, we examined several known proteins with E3 ligase activity. In cells transfected with TRAF-6 shRNA, the Id-1 and Id-2 protein levels were significantly increased (Fig. 6d, Supplementary Fig. 6c), and in the absence of TRAF-6 expression, less K63-linked polyubiquitination of Id-1 and Id-2 were observed (Fig. 6c). This finding further suggested that TRAF-6 was an E3 ubiquitin ligase involved in the K63-ubiquitination of Id-1 and Id-2.

As TRAF-6 could modulate the ubiquitination, we next wanted to determine whether Id-1 could bind to TRAF-6 and mapped the binding site of Id-1. Two deletion mutants of Id-1 could bind to TRAF-6 and have no obvious effect compared with the full-length Id-1. This suggested that it was the bHLH domain of Id-1 responsible for its binding to TRAF-6 (Fig. 6e). There were three lysine residues in the bHLH domain of human Id-1. We found that Lys88 was highly conserved among animalia (Fig. 6f). Thus, we speculated the Id-1 residue Lys88 may be a potential ubiquitylation site catalyzed by TRAF-6. To verify this hypothesis, the plasmid with Id-1 Lys88 residue mutated to arginine was constructed. The ubiquitination of Id-1 K88R was decreased even under the treatment of ionomycin, demonstrating that the Lys88 residue was essential for the Id-1 K63-ubiquitination stimulated by the ionomycin (Fig. 6g). Indeed, the Id-1 with deubiquitylated mutant was more stable than the wild-type one (Supplementary Fig. 8d, f). The results above indicated that Lys88 was the ubiquitination site of Id-1 that mediated this autophagic degradation.

### CaMKII phosphorylates Id-1 at Ser36

**Fig. 7:**
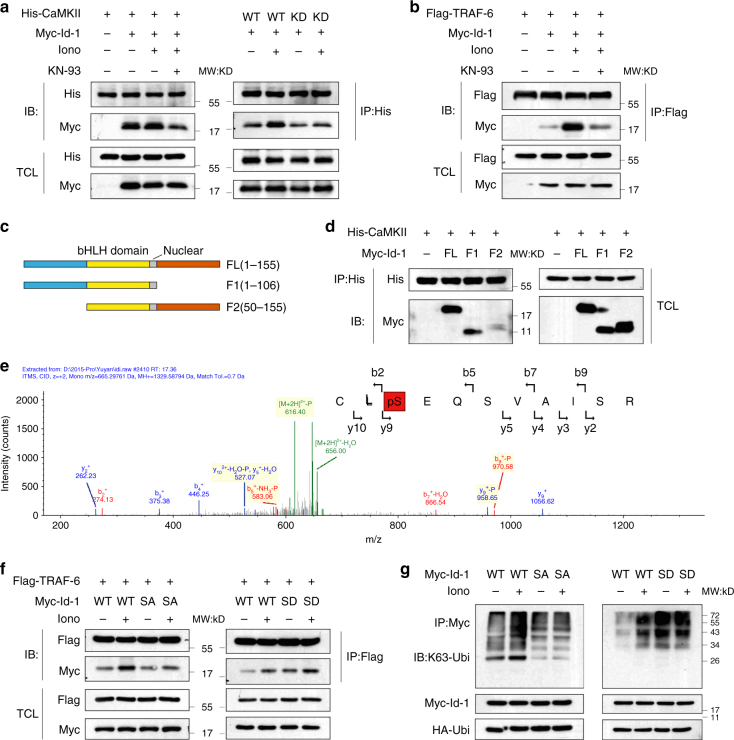
The phosphorylation of Ser36 is essential to the Lys-63-linked ubiquitylation of Id-1. **a** The interaction between Id-1 and CaMKII. HEK293T cells were transfected with the indicated plasmids for 24 h, incubated with 6 μM ionomycin or 10 μM KN-93 for 24 h. His-tagged proteins were precipitated and analyzed by western blot using the indicated antibodies. **b** The interaction between Id-1 and TRAF-6. HEK293T cells were transfected with the indicated plasmids for 24 h, and then treated with 6 μM ionomycin or 10 μM KN-93. The cells were treated with Baf A1 for 4 h before collect. The whole-cell lysates were analyzed by immunoblotting after precipitated by a Flag-M2 beads. **c** The illustration of Id-1 deletion constructs. **d** The 1–50 AAs fragment of Id-1 was essential to binding with CaMKII. HEK293 cells were co-transfected with vectors encoding CaMKII together with Myc-Id-1 FL (full-length) or its deletion mutants. Protein extracts were immunoprecipitated using anti-His antibody. **e** Tandem mass spectrometry analysis of Id-1 proteins after incubation with recombinant CaMKII in a kinase reaction. The data shown that the serine residue corresponding to Ser36 (indicated in red background in the peptide sequence) is phosphorylated. y, product ion numbered from C terminus of the peptide; b, product ion numbered from N terminus of the peptide. **f** The phosphorylation of Id-1 Ser36 was essential for the interaction of Id-1 and TRAF-6. HEK293T cells were transiently transfected with the indicated plasmids for 24 h and then treated with 6 μM ionomycin for 24 h and Baf A1 for 4 h before collect. The whole-cell extracts were subjected to western blotting using the indicated antibodies after an immunoprecipitation. **g** The phosphorylation of Id-1 Ser36 was required for the K63-ubiquitination of Id-1. HEK293T cells were transiently transfected with the indicated plasmids for 24 h and then treated with 6 μM ionomycin for 24 h and Baf A1 for 4 h before collect. The whole-cell extracts were subjected to western blotting using the indicated antibodies after an immunoprecipitation

It has been showed that the Lys-63-linked ubiquitylation of Ids was mediated by TRAF-6 and this modification could be enhanced with the treatment of ionomycin. To evaluate the connection between CaMKII and Id-1, we examined the interactions between them. Using immunoprecipitation, we observed that there was a basal level of association between CaMKII and Id-1 and this association increased after treatment with ionomycin (Fig. 7a). However, under the kinase dead CaMKII transfection or treatment with CaMKII inhibitior KN-93, the association was decreased (Fig. 7a). As shown in Fig. 7b, the interaction of Id-1 and TRAF-6 exhibited a similar tendency to the Id-1/CaMKII after treatment of ionomycin or KN-93, which revealed internal relation between the three.

To map the CaMKII-binding site on Id-1, two deletion mutants of Id-1 along with ectopic expression of His-CaMKII were used for the co-immunoprecipitation assays. The results showed that the fragment containing 1–50 amino acids but not the bHLH domain of Id-1 took a part in interaction with CaMKII (Fig. 7c, d).

Taken together, our results led us to propose a role for CaMKII as a kinase of Id proteins in regulating the K63-ubiquitylation of them and the subsequently autophagic degradation. Mass spectrometry analysis after an in vitro CaMKII kinase assay showed that Id-1 Ser36, which located in the CaMKII-binding fragment, could be phosphorylated by CaMKII (Fig. 7e). Furthermore, the in vitro kinase assay was performed by using an antibody against phospho-Ser, and the results indicated that wild-type Id-1 protein but not the Id-1-S36A mutant one could be phosphorylated by CaMKII directly (Supplementary Fig. 8e).

We next sought to confirm if the interaction between Id-1 and TRAF-6 was affected by the phosphorylation of Id-1. Myc-Id-1 with two kinds of point mutation plasmids to mimetic the phosphorylated (S36D) or dephosphorylated (S36A) serine were co-expressed with Flag-TRAF-6 in 293T cells. Indeed, the interaction between Id-1-S36D and TRAF-6 was enhanced (Fig. 7f, right). However, the interaction of Id-1-S36A and TRAF-6 was decreased, even with the treatment of ionomycin (Fig. 7f, left). We also examined the role of Ser36 phosphorylation on the K63-ubiquitylation of Id-1. As shown in Fig. 7g, the Id-1 K63-ubiquitylation was increased with the phosphorylated mutant (Id-1 S36D), but decreased with the dephosphorylated one (Id-1 S36A). However, when the Ser36 of Id-1 was mutant, ionomycin exposure could no longer elevate the subsequently ubiquitylation. Moreover, the S36A site mutant Id-1 proteins were more stable than the WT one (Supplementary Fig. 8d). Our conclusion, therefore, was that Id-1 phosphorylation at Ser36 promoted Id-1 K63-ubiquitylation by affecting the binding of Id-1 and TRAF-6.

### CaMKII induces the differentiation of neuroblastoma cells

**Fig. 8:**
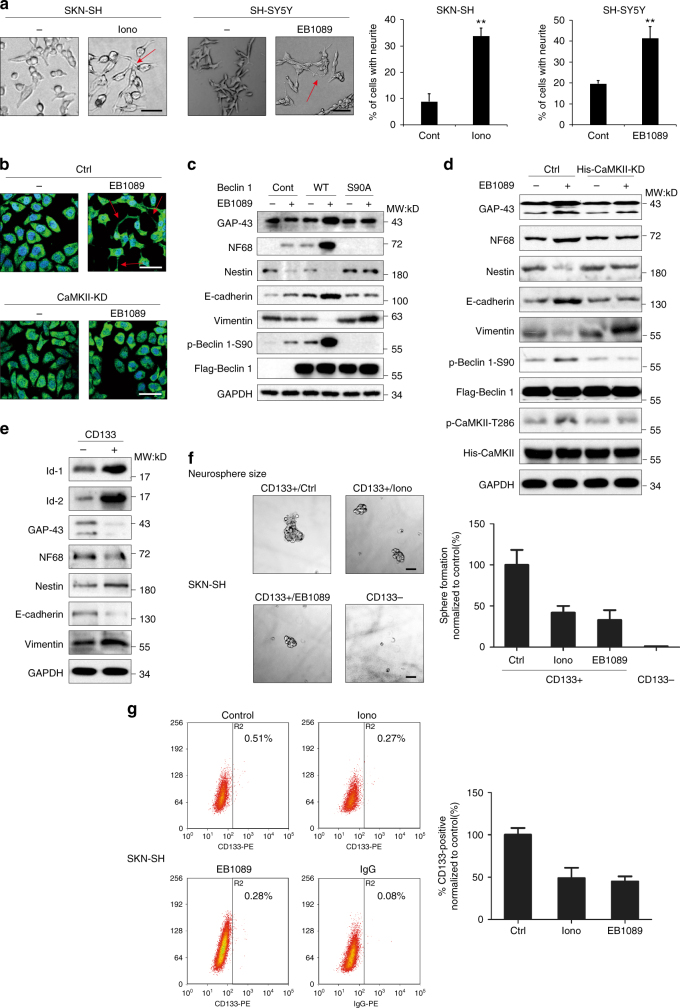
The CaMKII-mediated phosphorylation of Beclin 1 at Ser90 induces the differentiation of neuroblastoma cells. **a** Ionomycin and EB1089 regulate neuroblastoma cell differentiation. Both cell lines were treated with 1.5 μM ionomycin or 50 nM EB1089 for 7 days and then observed using fluorescence microscopy. Values were shown as the means ± SD of 5 random areas. **P < 0.01, Student’s *t*-test, Scale bar, 30 μm. **b** CaMKII knockdown impaired differentiation in neuroblastoma cells. SK-N-SH cells were transiently transfected with CaMKII-KD and treated with 100 nM EB1089 for 24 h. The cells were fixed and NF68 staining were analyzed using confocal fluorescence microscopy. Scale bar, 30 μm. **c** Phosphorylation of Beclin 1-S90 is required for EB1089-induced differentiation in neuroblastoma cells. SK-N-SH cells were transiently transfected with Vector control, Flag-Beclin 1-WT or Flag-Beclin 1-S90A plasmids and treated with 100 nM EB1089 for 24 h. The cell extracts were analyzed by western blot. **d** Activated CaMKII is required for EB1089-induced differentiation in neuroblastoma cells. SK-N-SH cells were transiently transfected with Control or His-CaMKII-KD plasmids and treated with 100 nM EB1089 for 24 h. The cell extracts were analyzed by western blot. **e** The expression of neuroblastoma cell differentiation-related molecules in CD133-positive cells and CD133-negative cells. Flow cytometric analysis of CD133 expression profiles in SK-N-SH cells. The CD133-positive cells and the CD133-negative cells were lysed, and the extracts were subjected to western blot using the indicated antibodies. **f** The inhibitory effect of ionomycin /EB1089 on neurosphere formation. CD133 expression profiles were analyzed by flow cytometry in SK-N-SH cells. The CD133-positive and CD133-negative cells were cultured in neurosphere-forming conditions. Neurospheres were incubated with 3 μM ionomycin or 100 nM EB1089 for 7 days. Treatment with ionomycin or EB1089 reduced the number and the size of neurospheres. Values were shown as the means ± SD. Scale bar, 100 μm. **g** The inhibitory effect of ionomycin and EB1089 on CD133-positive cells. SK-N-SH cells were untreated or treated with 3 μM ionomycin or 100 nM EB1089 for 24 h, and the CD133 expression profiles were analyzed by flow cytometry. CD133 fluorescence is depicted on the X-axis, and the percentage of CD133-positive cells is shown in the right-upper corner of each plot. Values were shown as the means ± SD calculated from three parallel experiments

The poor differentiation of neuroblastoma cell is often associated with the degree of its malignancy. The cells were treated with ionomycin, EB1089 or Vitamin D3 for 1 week; after this period, the cells were found to elongate a large number of well-developed neurite appeared between the cell junctions (Fig. 8a, Supplementary Fig. 10a). This was one distinct morphological characteristic of tumor cell differentiation. To further identify the role of CaMKII on the differentiation of neuroblastoma cells, we stained SK-N-SH cells with NF68, a neurofilament marker, and then measured the cellular morphology (Fig. 8b). The formation of neurite was observed in cells treated with EB1089; however, the CaMKII-KD mutant severely reduced the number of cellular neurite. Previous research has claimed that the high expression of the Id proteins can significantly inhibit cell differentiation^42^. To verify this statement, we knocked down Id-1 or Id-2 with shRNA in neuroblastoma cells. Consistent with previous findings, when the expression of the Id proteins was silenced, neurite became longer, suggesting more marked cell differentiation (Supplementary Fig. 12f). Furthermore, in IMR-32 cells with high Id expression levels, the percentage of CD133-positive cells decreased more rapidly in response to ionomycin treatment. However, the SK-N-BE(2) cell line with low Id expression showed less change of CD133^+^ proportion (Supplementary Fig. 12d, e).

Next, the expression levels of several neuroblastoma-related differentiation protein markers were examined. After EB1089 treatment, the expression of GAP43, NF68, and E-cadherin was increased, while Nestin and Vimentin were downregulated, suggesting that EB1089 induces differentiation of SK-N-SH cells (Fig. 8c). In SK-N-SH cells that were transfected with the Flag-Beclin 1-WT plasmid, the effect of EB1089 was enhanced, but with the Flag-Beclin 1-S90A plasmid, EB1089 failed to induce cell differentiation (Fig. 8c, Supplementary Fig. 10d, e). Also cell differentiation was impaired by CaMKII-KD or Atg5 siRNA (Fig. 8d and Supplementary Fig. 10b, c). Moreover, overexpression of CaMKII-CA plasmid promoted cellular differentiation, which was abrogated by Beclin 1-S90A but not Beclin 1-WT (Supplementary Fig. 9a). These findings strongly suggested that the EB1089-induced differentiation depends on the phosphorylation of Beclin 1 at Ser90 and the autophagy activity.

In addition, CD133, a marker for stem cell-like neuroblastoma cells, was used to sort the SK-N-SH cells^43^. The proportion of stem cells among the SK-N-SH cells was maintained at ~0.5%, and our results showed that the CD133-positive cells displayed neuroblastoma stem-like cell properties (Fig. 8e).

CD133-positive cells were then subjected to suspension culture and exposed to ionomycin or EB1089 treatment. We found that the size and number of cellular spheres were significantly inhibited (Fig. 8f), indicating that ionomycin and EB1089 can reduce the proportion of stem cells by inducing the differentiation of stem cell-like neuroblastoma cells. To further clarify whether ionomycin/EB1089-induced differentiation was correlated with the phosphorylation of Beclin 1, several CaMKII-related plasmids were used. As shown in Supplementary Fig. 9b–d, CaMKII knockdown reverted the size of cellular spheres, as same as CaMKII-KD. On the contrary, cellular spheres of cells expressing activated CaMKII were markedly decreased.

Finally, in the cells that were exposed to ionomycin, EB1089 or Vitamin D3, the proportion of stem cell-like cancer cells in various groups of cells was determined using CD133 as the stem cell marker. Consistent with the results from the suspension sphere cultures, both agents reduced the proportion of stem cells in neuroblastoma cells (Fig. 8g, Supplementary Fig. 12a). In addition, CaMKII-KD and Beclin 1-S90A could reverse the diminution of stem cells’ proportion in response to ionomycin (Supplementary Fig. 11a, b), so did the Id-1-S36A and Atg5 shRNA (Supplementary Fig. 11c, d). Furthermore, phosphorylation of Beclin 1-S90 together with CD133 expression was detected in four different neuroblastoma cell lines, and the result shows a negative relation (Supplementary Fig. 12b, c). These results provide a theoretical basis for the clinical application of ionomycin or EB1089.

## Discussion

**Fig. 9:**
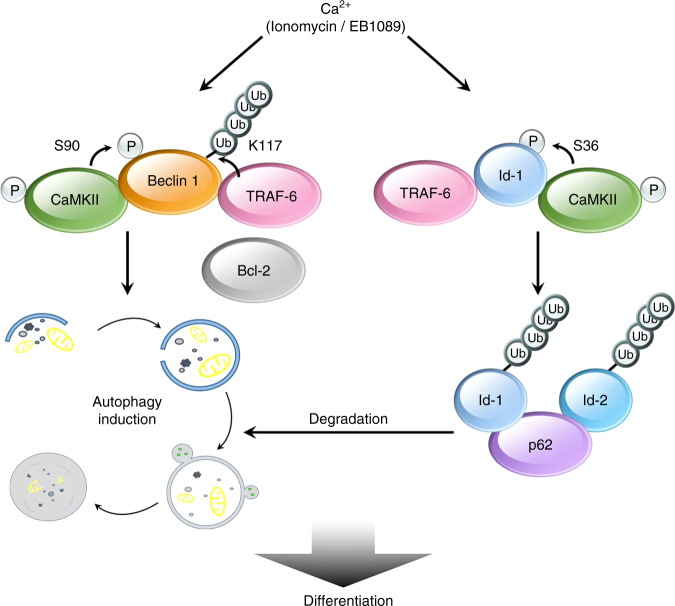
Model of Beclin 1 and Ids regulation by CaMKII. Proposed model for CaMKII-mediated regulation of Beclin 1 and Id proteins

Our study demonstrates for the first time that CaMKII can directly associates with Beclin 1 and phosphorylates its Ser90 site, promotes the K63-linked ubiquitination of its Lys117 site, thereby activating the autophagy pathway. CaMKII induces the phosphorylation of Id-1, which is associated with the involvement of TRAF-6 and the K63-linked ubiquitination of Id-1. Id-1/Id-2 further binds to p62, and be transported to autolysosomes for degradation, thus regulating the differentiation of tumor cells and reducing the proportion of stem cell-like cancer cells (Fig. 9).

Although, it has been understood that there is a relationship between autophagy and cancer, the progress has only been made in the understanding of the molecular links between autophagy and cancer recently. On one hand, autophagy can promote tumor cell survival and contribute to cancer tolerance to drug therapy under certain conditions^44, 45^; on the other hand, autophagy can inhibit tumorigenesis. Defective autophagy can lead to the accumulation of intracellular damage. In some cases, autophagy is also involved in drug-induced tumor cell death^46, 47^. In short, autophagy is implicated throughout the process of tumor development and progression. In-depth studies of autophagy genes may open up new avenues for the prevention and treatment of malignant tumors.

In dissecting the regulatory mechanism of Beclin 1 and its role in tumors, we found that calcium-related pathways are involved. Much of the research effort has been focused on the Ca^2+^-CaMKKβ-AMPK-mTOR pathway, whose effect on autophagy is also reportedly dependent on Beclin 1^31^. This pathway indirectly affects Beclin 1 to induce autophagy. Our findings showed that CaMKII directly phosphorylates Beclin 1 at Ser90 to induce autophagy. This represents a new regulatory mechanism involving Beclin 1. In our present study, we noted that when CaMKII failed to phosphorylate Beclin 1, autophagy was reduced but not completely abolished, suggesting that there are other molecular mechanisms by which ionomycin can regulate autophagy; this is consistent with other reports.

Autophagy is a conservative degradation pathway in eukaryotic cells. Stress-induced protein aggregation can also be degraded through autophagy. Many proteins aggregate in autophagy-deficient cells, suggesting that autophagy plays an important role in the control of intracellular protein metabolism and that it cannot be fully replaced by proteasome-mediated protein degradation. Our study showed that while CaMKII activated the autophagy pathway by phosphorylating Beclin 1, CaMKII promoted Id-1/Id-2 ubiquitination, targeting two inhibitor of differentiation proteins to autophagic degradation.

Recent studies have shown that the Id proteins are highly expressed in many solid tumors and correlate with the degree of tumor differentiation^48–50^. Therefore, the degradation of Ids may promote the differentiation of tumor cells. Much progress has been made in the induction of tumor cell differentiation over the past decade. Inducing the differentiation of malignant tumors has opened up a very promising avenue for the treatment of malignant tumors. However, very few studies have reported successful treatment with induced differentiation of solid tumors. Further studies are warranted to gain more insight into the underlying molecular mechanism. We found that ionomycin/EB1089 can activate CaMKII to phosphorylate Beclin 1/Id-1, which further induces the differentiation of neuroblastoma cells. Meanwhile, both ionomycin and EB1089 can effectively reduce sphere formation and reduce the proportion of stem cell-like neuroblastoma cells. These results provide a theoretical basis for further clinical application of the drugs. Cancer stem cells have a broad differentiation potential and contribute to tumor development and growth, and our present study showed that reducing the proportion of cancer stem cells by promoting cancer stem cell differentiation is of great significance for controlling tumor recurrence and metastasis.

In summary, our study provides further insight into the impact of Beclin 1 phosphorylation on the autophagy potential of tumor cells and presents evidence for the role of autophagy induced by CaMKII phosphorylation of Beclin 1 in solid tumor cell differentiation. Autophagy-targeting drugs have emerged as a hotspot in cancer research. Our research provides a basis and guidance for autophagy-targeting drugs and their application in cancer therapy, and further clarifies the role and regulation of Id proteins in solid tumor cell differentiation, offering new targets and ideas for cancer therapy.

## Methods

### Cell cultures

HEK293T, SK-N-SH, SH-SY5Y, and HeLa cell lines were ordered from American Type Cell Culture (Manassas, VA). The human neuroblastoma cell lines SK-N-BE(2), IMR-32 were purchased from the Shanghai Institutes for Biological Sciences. All the cell lines have been authenticated by the corresponding Institutes where we purchased from. The human neuroblastoma cell lines SK-N-SH and SH-SY5Y were grown in DMEM medium supplemented with 10% FBS (heat inactivated at 56 °C for 30 min) and the appropriate amount of penicillin (50 U ml^−1^)/streptomycin (50 mg ml^−1^) in a 37 °C incubator with a humidified 5% CO_2_ atmosphere.

### Antibodies and chemicals

Antibodies specific for Beclin 1 phosphorylated on Ser90 were generated by Abgent (San Diego, CA, USA, 1:500). Other primary antibodies used for western blotting were anti-Beclin 1 (Cell Signaling Technology, #3738, 1:1000), anti-GAPDH (KangChen, Shanghai, China, 1:10,000), anti-p-CaMKII (Cell Signaling Technology, #3361, 1:1000), anti-CaMKII (Santa Cruz, sc-1541, 1:500), anti-Bcl-2 (Santa Cruz, SC-7382, 1:500), anti-LC3 (Novus, Littleton, CO, USA, NB100-2220, 1:3000), anti-Id-1 (Santa Cruz, sc-488, 1:1000), anti-Id-2 (Cell Signaling Technology, #3431, 1:500), anti-SQSTM1 (Santa Cruz, sc-28359, 1:1000), anti-His-Tag (Cell Signaling Technology, #2366, 1:1000), anti-Myc-Tag (Cell Signaling Technology, #2276, 1:1000), anti-Flag (Sigma-Aldrich, F1804, 1:2000), anti-ubiquitin (Santa Cruz, sc-58449, 1:1000), anti-K63-linkage-specific polyubiquitin (Cell Signaling Technology, #5621, 1:1000), anti-TRAF-6 (Cell Signaling Technology, #8028, 1:1000), anti-GAP43 (Cell Signaling Technology, #8945 s, 1:1000), anti-NF68 (Cell Signaling Technology, #2837 s, 1:1000), anti-nestin (Santa Cruz, SC-23927, 1:1000), anti-vimentin (BD, 550513, 1:1000), and anti-E-cadherin (BD, 51-9001922, 1:1000). KN-93, MG132, and bafilomycin A1 were purchased from Sigma-Aldrich. Ionomycin was purchased from Cell Signaling Technology. EB1089 was purchased from Santa Cruz.

### Western blotting and immunoprecipitation

Cells were solubilized in lysis buffer (Cell Signaling Technology) containing Complete Protease Inhibitor Cocktail (Roche Applied Sciences). The protein concentration was determined using the Pierce BCA protein assay kit (Thermo). Equal amounts of proteins were electrophoretically separated in SDS-polyacrylamide gels and transferred to PVDF membranes (Millipore). The membranes were then blocked in a 5% skim milk solution in Tris-buffered saline containing 0.1% Tween (TBST) for 2 h and incubated at 4 ˚C overnight with a primary antibody. Immunoreactivity was detected using the Amersham ECL Prime western blotting detection reagent (GE Healthcare, Piscataway, NJ, USA) according to the manufacturer’s instructions. For immunoprecipitation, cells were collected and lysed in Radio Immunoprecipitation Assay (RIPA) Lysis Buffer (Millipore) supplemented with Complete Protease Inhibitor Cocktail (Roche). After preclearing with protein A/G agarose (Roche) beads for 1 h at 4 °C, whole-cell lysates were used for immunoprecipitation with the indicated antibodies. Generally, 1–4 μg of commercial antibody was added to 1 ml of cell lysate, and the mixture was incubated at 4 °C for 1 h. After adding protein A/G agarose beads, the incubation was continued for 1 h or overnight. Immunocomplexes were extensively washed four times with RIPA buffer, heated to 95 ˚C for 3 min and separated by SDS-PAGE. Original blot images are shown in Supplementary Figs. 13, 14.

### Plasmids

The plasmid encoding human Beclin 1 was purchased from OriGene (Rockville, MD, USA). The plasmid encoding human Id-1 and Id-2 were purchased from GenChem (Changzhou, China). The HA-Ubiquitin construct was kindly provided by Professor Kang (State Key Laboratory of Oncology in South China; Cancer Centre, Sun Yat-sen University, Guangzhou, China). The deletion mutants of Id-1 were constructed by subcloning GenChem plasmid into pcDNA3 vector. To obtain the sequences of Id-1, the PrimeSTAR Max DNA Polymerase (Takara, #R045Q) was used, then the PCR products and vector plasmid were cut by double restriction endonuclease. The PCR products and vector were linked by T4 DNA ligase (Takara, #2011A). Mutations were introduced using the QuikChange site directed mutagenesis kit (Stratagene, Santa Clara, CA, USA), and all mutations were verified by DNA sequencing.

### shRNA

Short hairpin RNA (shRNA) sequence against Beclin 1 was purchased from OriGene. The target sequence for the TRAF-6-specific shRNA is 5′-GCCACGGGAAATATGTAATATCT-3′, and the target sequence for Id-2-specific shRNA is 5′-AAGCACTGTGTGGCTGAATAA-3′. The CaMKII, TRAF-6, Id-2, and control (no silencing) shRNAs were synthesized by GenChem. Transfection was performed using the Lipofectamine 2000 transfection reagent according to the manufacturer’s instructions (Invitrogen).

### RNA extraction and real-time RT-PCR

Total RNA from cultured cells was extracted using the TRIzol reagent (Invitrogen) according to the manufacturer’s instructions. The RNA was pretreated with RQ1 RNase-free DNase (Promega, Madison, WI, USA), and 2 μg RNA from each sample was used for cDNA synthesis, which was primed using an oligo-(dT) 18 primer (TaKaRa, Dalian, China). For Beclin 1 and GAPDH real-time RT-PCR, we used SYBR Green qPCR primer pairs (OriGene). To detect Beclin 1 mRNA, the following real-time RT-PCR primers were used: forward primer: 5′-GCTCCCGAGGTGAAGAGCAT-3′ and reverse primer: 5′-GCCTGGGCTGTGGTAAGTAA-3′. The primers 5′-AAGCCTGCCGGTGACTAAC-3′(forward) and 5′-GTTAAAAGCAGCCCTGGTGAC-3′ (reverse) were used to generate a 174-bp GAPDH fragment as an internal control. To ensure reproducibility, all genes were assayed in triplicate. The real-time RT-PCR reactions were performed using the following cycling parameters on an ABI PRISM 7900HT sequence detection system: an initial denaturing step at 95 ˚C for 10 min, followed by 40 cycles of 95 ˚C for 15 s, 60 ˚C for 10 s, and 72 ˚C for 25 s.

### Kinase assays

The full-length WT and Ser90 mutant Beclin 1 fusion proteins, the full-length WT Id-1 fusion protein were produced by OriGene. Active CaMKII was purchased from Sigma-Aldrich. The kinase reactions were performed in the buffer recommended by the manufacturer supplemented with 500 mM ATP, 5 μCi of [γ-^32^P] ATP, 2 μg of soluble Beclin 1, and 100 ng of CaMKII. The reaction was incubated at 30 °C for 1 h and then resolved by SDS-PAGE followed by western blot and autoradiography.

### Mass spectrometry

The Flag-Id-1 fusion protein was excised from a SDS-PAGE gel after an in vitro kinase assay. The Id-1 band was subjected to trypsin digestion and the liquid chromatography coupled tandem mass spectrometry. Protein and modification identification was performed with the database search and peptide identifications were validated with peptide prophet.

### Immunofluorescence

The cells were plated at 50% confluence on glass coverslips. After the tumor cells were treated as previously described, the cells were washed briefly with PBS, fixed for 30 min at room temperature in 4% paraformaldehyde, blocked in a 4% BSA solution in PBS for 1 h and incubated at 4 ˚C overnight with a primary antibody. The specimens were then incubated in fluorochrome-conjugated secondary antibody for 1 h at room temperature in the dark followed by 1 μg ml^−1^ DAPI (Sigma-Aldrich) for 5 min, washed with PBS and dried. The cells were observed at ×100 magnification using an Olympus FV-1000 confocal microscope.

### Sorting of CD133^+^ cells by flow cytometry

Cells were analyzed by FACS when the cells had reached a logarithmic growth phase (24 h after replating). The cells were digested with 0.25% trypsin, washed twice with calcium/magnesium-free PBS, resuspended in PBS at a concentration of 1 × 10^6^cells per mL. A CD133/1(AC133)-PE antibody (Bergisch Gladbach, Germany, #130-080-801) was then added to the cell suspension at the ratio of 1:11 (V/V) and incubated in the dark at 4 °C for 10 min. The cells were then washed twice with PBS and kept at 4 °C in the dark prior to analysis by a MofloXDP (Beckman Coulter, CA, USA).

### Neurosphere formation assay

Neurosphere culture was performed in serum-free DMEM-F12 medium (Invitrogen) supplemented with 2% B27 (Invitrogen), 1% FBS, 1% N_2_ (Life Technologies, Grand Island, NY, USA), 20 ng mL^−1^ EGF (Sigma-Aldrich) and 10 ng mL^−1^ basic fibroblast growth factor (Sigma-Aldrich). Single cells were prepared from flow cytometry, trypsinized, and then plated in six-well ultralow attachment plates (Corning, NY, USA) at a density of 500–1000 cells per mL in primary culture. The second passages were grown in the absence of drugs. After 7 days of culture, the number of neurospheres was counted using a Nikon Eclipse TE2000-S microscope.

### Statistical analyses

The differences between two groups were assessed by Student’s *t*-test. *p < 0.05 was considered statistically significant; **p < 0.01 as highly significant.

### Data availability

The data that support the findings of this study are available from the corresponding author upon reasonable request.

## Electronic supplementary material

Supplementary Information
