# Supplementary material for: CaMKII-mediated Beclin 1 phosphorylation regulates autophagy that promotes degradation of Id and neuroblastoma cell differentiation
Source: Nat Commun. 2017 Oct 27;8:1159. doi: 10.1038/s41467-017-01272-2 (PMC5660092; doi:10.1038/s41467-017-01272-2)

Supplementary Figure 1 CaMKII activity affects the phosphorylation of Beclin 1 at Ser90

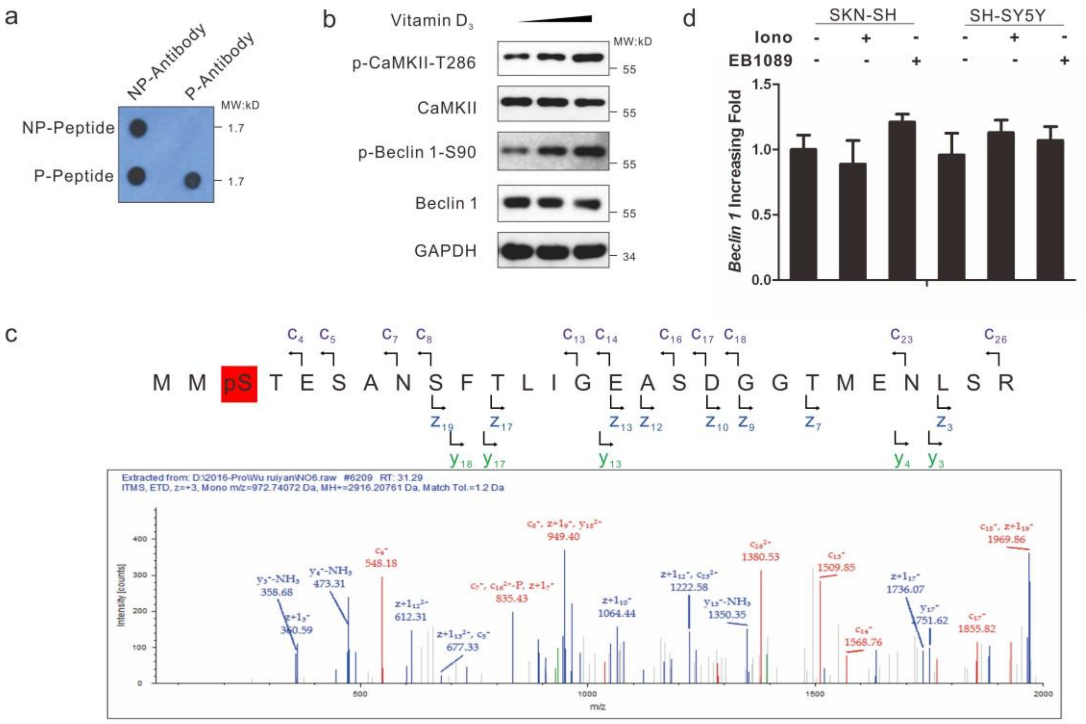

(a) The dolt blot report of p-S90 antibody produced by Abgent Biotechnology.

(b) Vitamin D3 regulates CaMKII activity and the phosphorylation of Beclin 1 Ser90. SK-N-SH cells were treated with 5, 10 μM Vitamin D3 for 24 h. The cell extracts were analyzed by Western blotting.

(c) Tandem mass spectrometry analysis of Beclin 1. 293 cells were co-transfected with Flag-Beclin 1 and His-CaMKII; The protein extracts were incubated with flag affinity gel and performed for mass spectrometry. It was shown that the serine residue corrresonding to Ser90 (indicated in red background in the peptide sequence) is phosphorylated. Y and z, product ion numbered from C terminus of the peptide; c, product ion numbered from N terminus of the peptide.

(d) Beclin 1 mRNA expression was unaffected in cells treated with ionomycin or EB1089. Both of the cell lines were untreated or were treated with 6μM ionomycin or 100 nM EB1089 for 24h. The expression level of Beclin-1 mRNA was detected by real-time RT-PCR. The error bars represent the standard deviations (SD) calculated from three parallel experiments.

Supplementary Figure 2 CaMKII induces autophagy through phosphorylation of Beclin 1 at Ser90

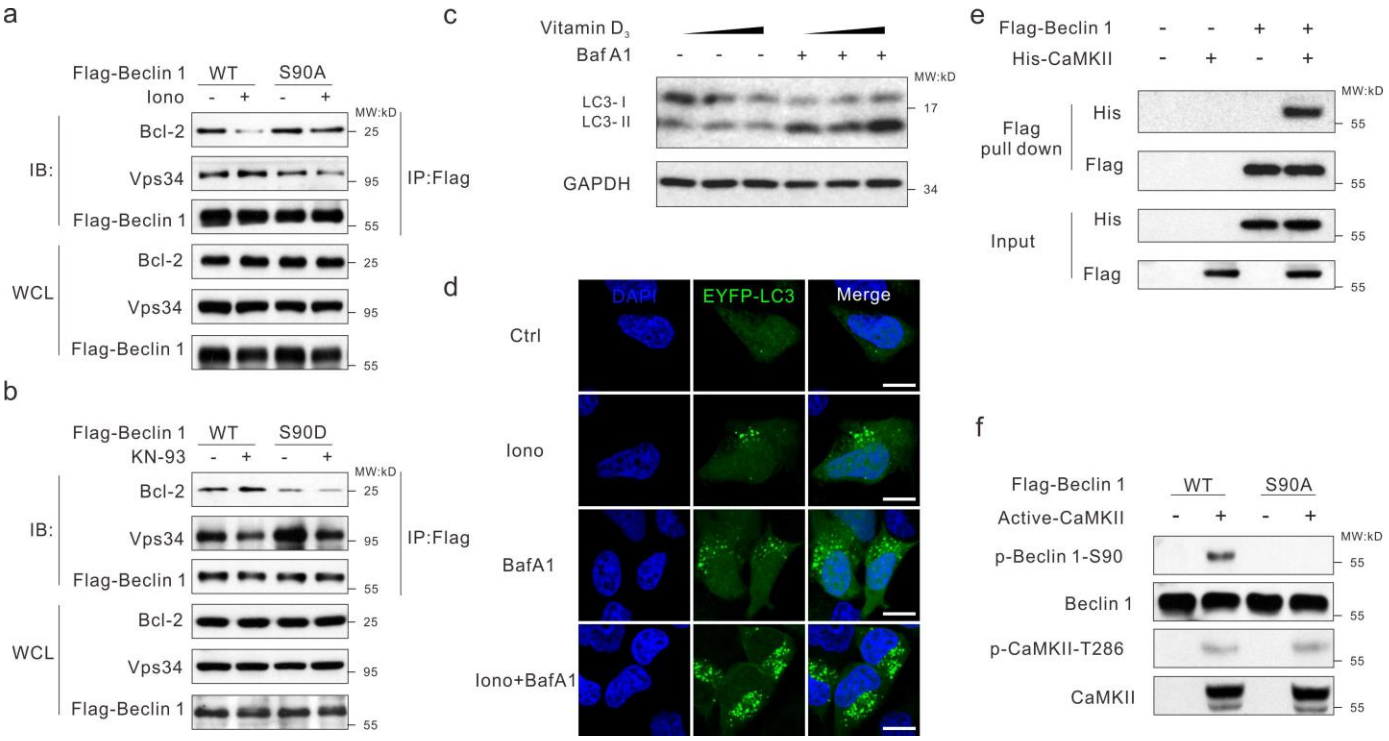

(a) The interaction between Beclin 1 and Beclin 1-binding proteins in Beclin 1-S90A expressing cells. HEK293T cells were transfected with Flag-Beclin 1 or Flag-Beclin 1-S90A plasmids, then treated with 6 μM ionomycin for 24 h. The cells were lysed and incubated with an anti-Flag antibody prior to Western blot. The resulting immunoprecipitates were subjected to Western blot analysis. WCL, the whole-cell lysate.

(b) The interaction between Beclin 1 and Beclin 1-binding proteins in Beclin 1-S90D expressing cells. HEK293T cells were transfected with Flag-Beclin 1 or Flag-Beclin 1-S90D plasmids and treated with 10 μM KN-93 for 24 h. The cells were lysed and incubated with an anti-Flag antibody. The resulting immunoprecipitates were subjected to Western blot analysis. WCL, the whole-cell lysate.

(c) Autophagy induced by Vitamin D3 in neuroblastoma cells. SK-N-SH cells were treated with 5/10 μM ionomycin for 24 h, then incubated with 100 nM BafA1 for 2h. The total cell lysates were analyzed for LC3 lipidation by immunoblotting.

(d) Autophagosome formation in Hela cells. The cells were incubated in 6 µM ionomycin for 24 h and 100 nM Baf A1 for 2h, and then fixed. Scale bars, 10 µm.

(e) The in vitro binding assay of Beclin 1 and CaMKII. Purified Flag-Beclin 1 and His-CaMKII proteins were incubated with Flag affinity gel for 24h at 4°C. The Pull-down mixture was subjected to Western blot analysis.

(f) *In vitro* phosphorylation of Beclin 1 by active CaMKII. Purified Beclin 1 fusion proteins were incubated with recombinant active CaMKII kinase, and *in vitro* kinase assays were performed. Reaction products were run on SDS-PAGE and subjected to Western Blot.

Supplementary Figure 3 CaMKII induces autophagy through phosphorylation of Beclin 1 at Ser90

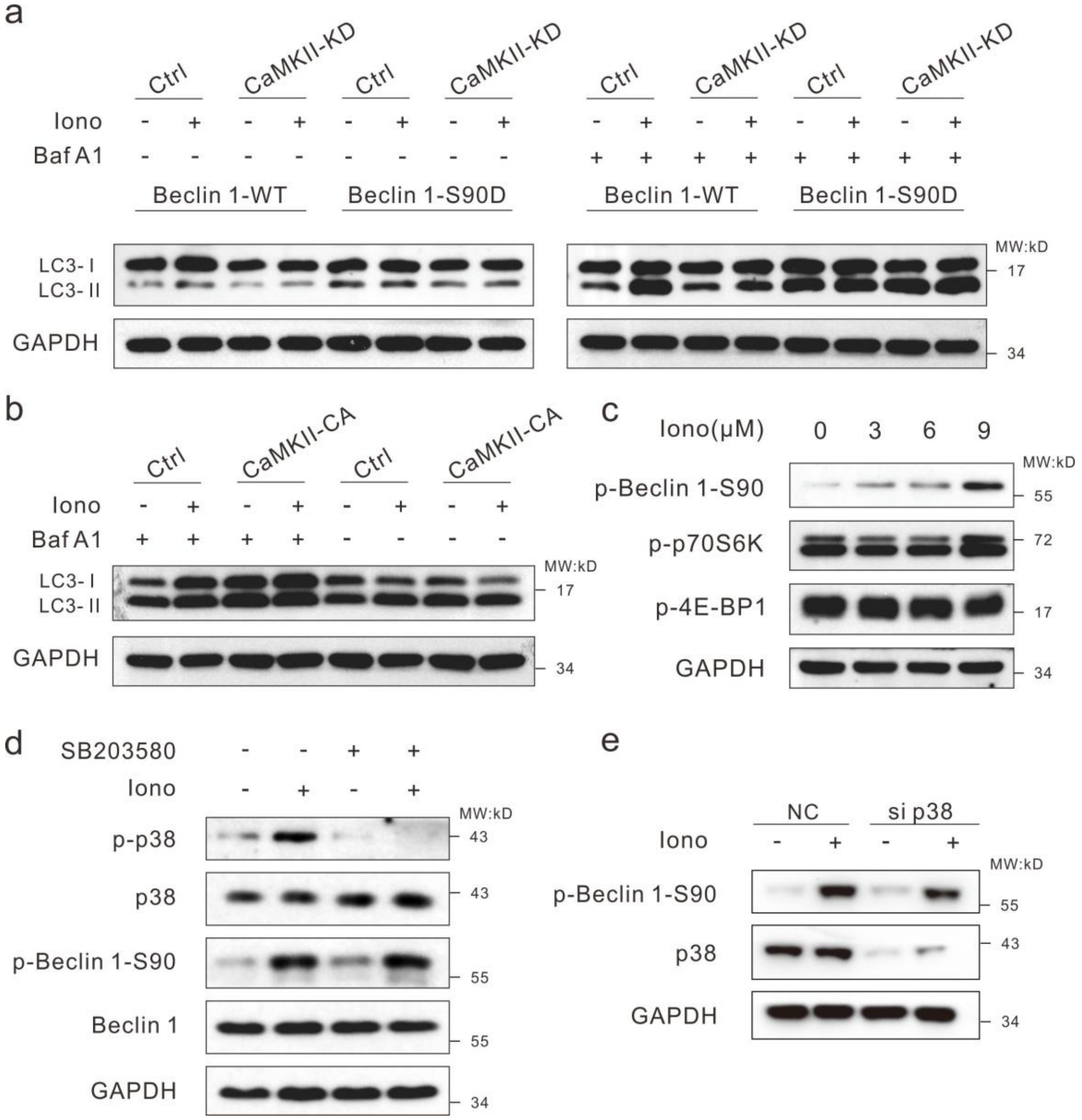

(a) LC3 lipidation decreasing in CaMKII-KD-expressing MEFs cells or control cells that can be restored by Beclin 1-S90D mutant. MEFs cells transfected with indicated plasmids were treated with 6 μM ionomycin for 24 h, then incubated with or without 100 nM BafA1 for 2h. The whole-cell extracts were subjected to Western blot using the indicated antibodies.

(b) LC3 lipidation in CaMKII-CA-expressing MEFs cells or control cells (Ctrl). MEFs cells transfected with a control vector or plasmids encoding His-CaMKII-CA were treated with 6 μM ionomycin for 24 h, then incubated with 100 nM BafA1 for 2h as indicated. The whole-cell extracts were subjected to Western blot using the indicated antibodies.

(c) mTOR pathway was not involved in Ionomycin-induced Beclin 1 phosphorylation. SK-N-SH cells were treated with various concentrations of ionomycin for 24 h. The cell extracts were analyzed by Western blotting.

(d) P38-MAPK was not involved in ionomycin-induced autophagy. SK-N-SH cells were treated with ionomycin or SB203580 for 24 h. The cell extracts were analyzed by Western blotting.

(e) P38-MAPK was not involved in ionomycin-induced autophagy. SK-N-SH cells were transiently transfected with the p38 siRNA and then treated with 6 μM ionomycin for 24 h. The cell extracts were analyzed by Western blotting.

Supplementary Figure 4 MAPKAPKs were not involved in ionomycin-induced Beclin 1 phosphorylation

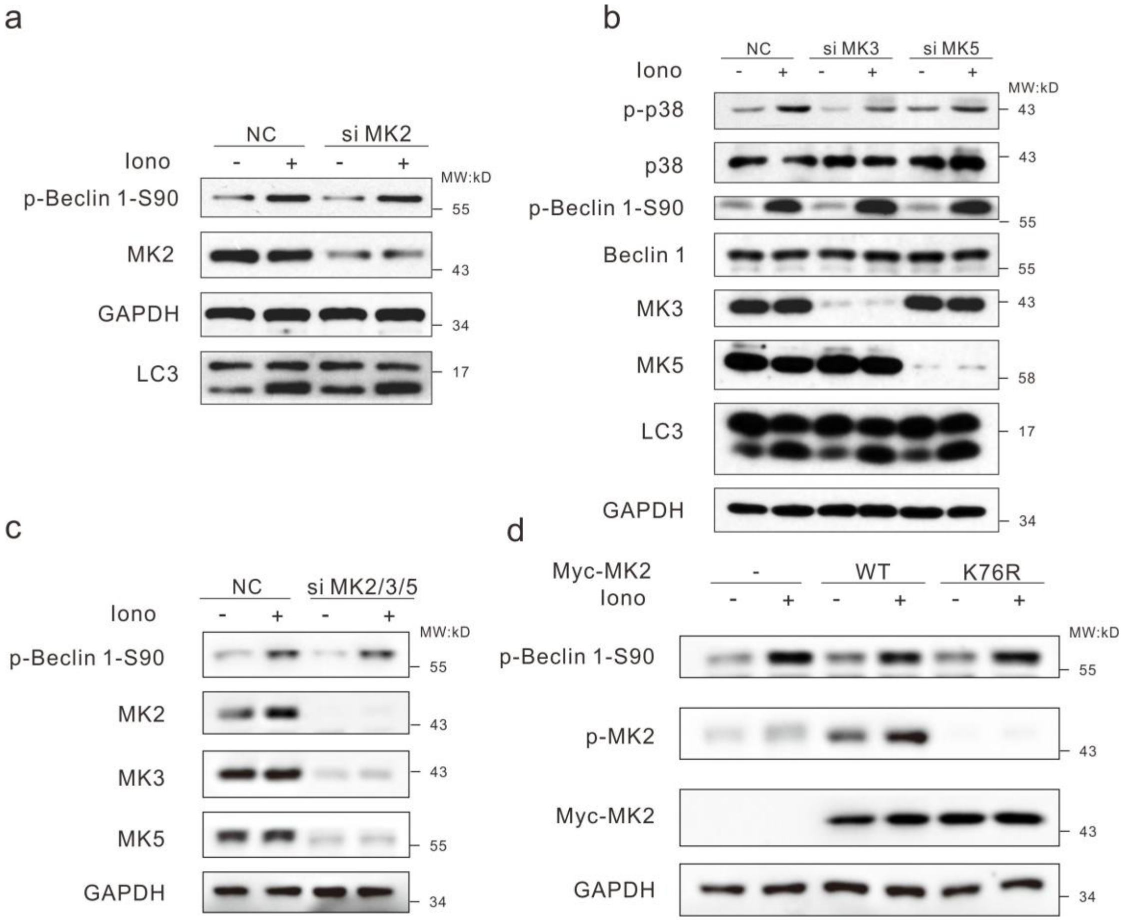

(a) MK2 was not involved in ionomycin-induced Beclin 1 phosphorylation. SK-N-SH cells were transiently transfected with the MK2 siRNA and then treated with 6 μM ionomycin for 24 h. The cell extracts were analyzed by Western blotting.

(b) MK3/5 was not involved in ionomycin-induced autophagy. SK-N-SH cells were transiently transfected with the MK3/5 siRNA and then treated with 6 μM ionomycin for 24 h. The cell extracts were analyzed by Western blotting.

(c) MKs was not involved in ionomycin-induced autophagy. SK-N-SH cells were transiently transfected with the MKs siRNAs at a same time and then treated with 6 μM ionomycin for 24 h. The cell extracts were analyzed by Western blotting.

(d) The activity of MKs didn't affect Beclin 1 phosphorylation. SK-N-SH cells were transiently transfected with the indicated plasmids and then treated with 6 μM ionomycin for 24 h. The cell extracts were analyzed by Western blotting.

Supplementary Figure 5 Ser90 phosphorylation is required for TRAF-6 mediated K63-ubiquitination of Beclin 1.

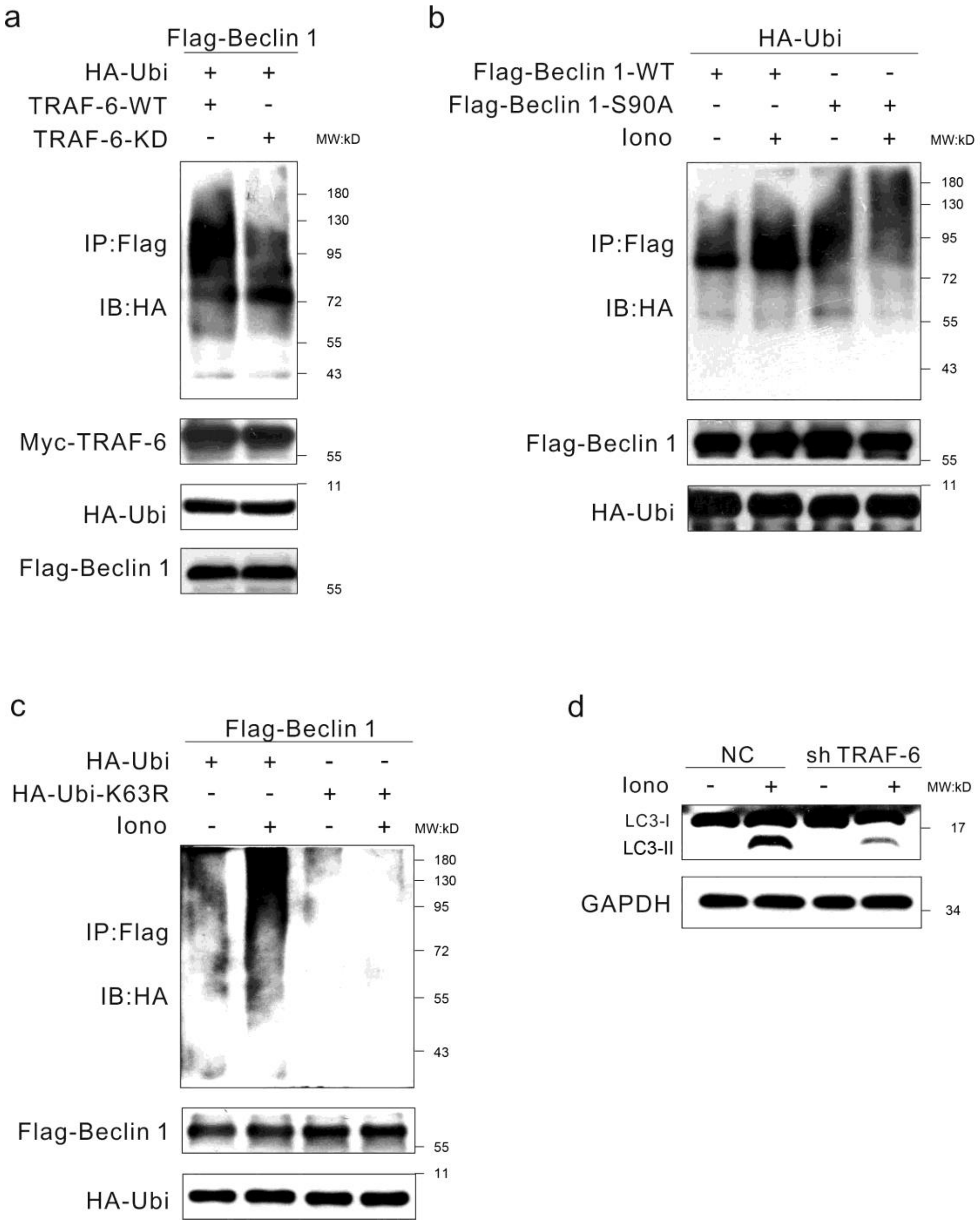

(a) TRAF-6 is required for the ubiquitination of Beclin 1. HEK293T cells were transfected for 24 h with the WT-TRAF-6 or KD-TRAF-6 for 48 h, then the immunoprecipitated Flag-Beclin 1 was subjected to anti-HA Western blot.

(b) The phosphorylation at S90 promoted ubiquitination on Beclin 1. HEK293T cells were transiently co-transfected with the indicated plasmids, and then treated with 6 μM ionomycin for

24 h. The immunoprecipitated Flag-Beclin 1 was subjected to anti-HA Western blot.

(c) Beclin 1 undergoes K63 ubiquitination. HEK293T cells were transiently co-transfected with the indicated plasmids, treated with 6 μM ionomycin for 24 h. The immunoprecipitated Flag-Beclin 1 was subjected to anti-HA Western blot.

(d) TRAF-6 is required in autophagy induced by ionomycin. HEK293T cells transfected with a negative control or TRAF-6 shRNA were treated with 6 μM ionomycin for 24 h, then incubated for 2 h in the presence or absence of 100 nM Baf A1. The whole-cell extracts were subjected to Western blot using the indicated antibodies.

Supplementary Figure 6 Autophagy induced by ionomycin promotes degradation of inhibitor of differentiation proteins

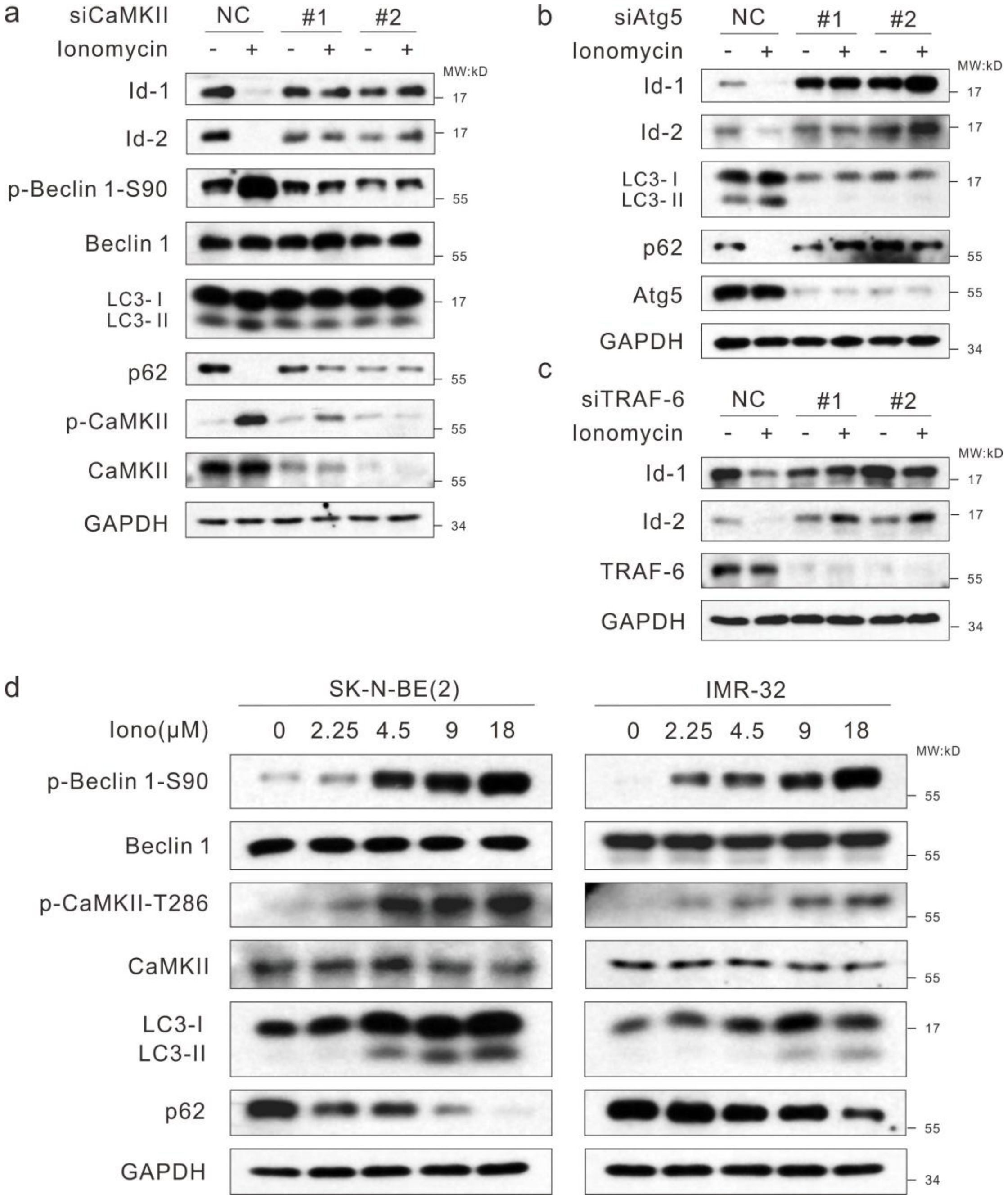

(a) CaMKII is required for phosphorylation of Beclin 1-S90, autophagy induction and the degradation of Id-1/2. HEK293T cells were transiently transfected with negative control or CaMKII siRNA, and then untreated or treated with 6 μM ionomycin for 24 h. The cells were lysed and the indicated proteins were analyzed by Western Blot.

(b) Id-1/2proteins are degraded via the autophagy pathway. SK-N-SH cells were transfected for 24 h with a negative control (NC) or Atg5 siRNA and then incubated for 24h with 6μM ionomycin.

The cell lysates were then analyzed by Western blotting.

(c) TRAF-6 is required for the degradation of Id-1/2. HEK293T cells were transfected for 24 h with the negative control (NC) or TRAF-6 siRNA and then incubated for 24 h with 6 μM ionomycin or 100 nM EB1089. The cell lysates were then analyzed by Western blot.

(d) Ionomycin induced phosphorylation of Beclin 1 and autophagy flux in other two neuroblastoma cell lines.

Supplementary Figure 7 Lys117 ubiquitilation of Beclin 1 is essential for Ionomycin-induced autophagy.

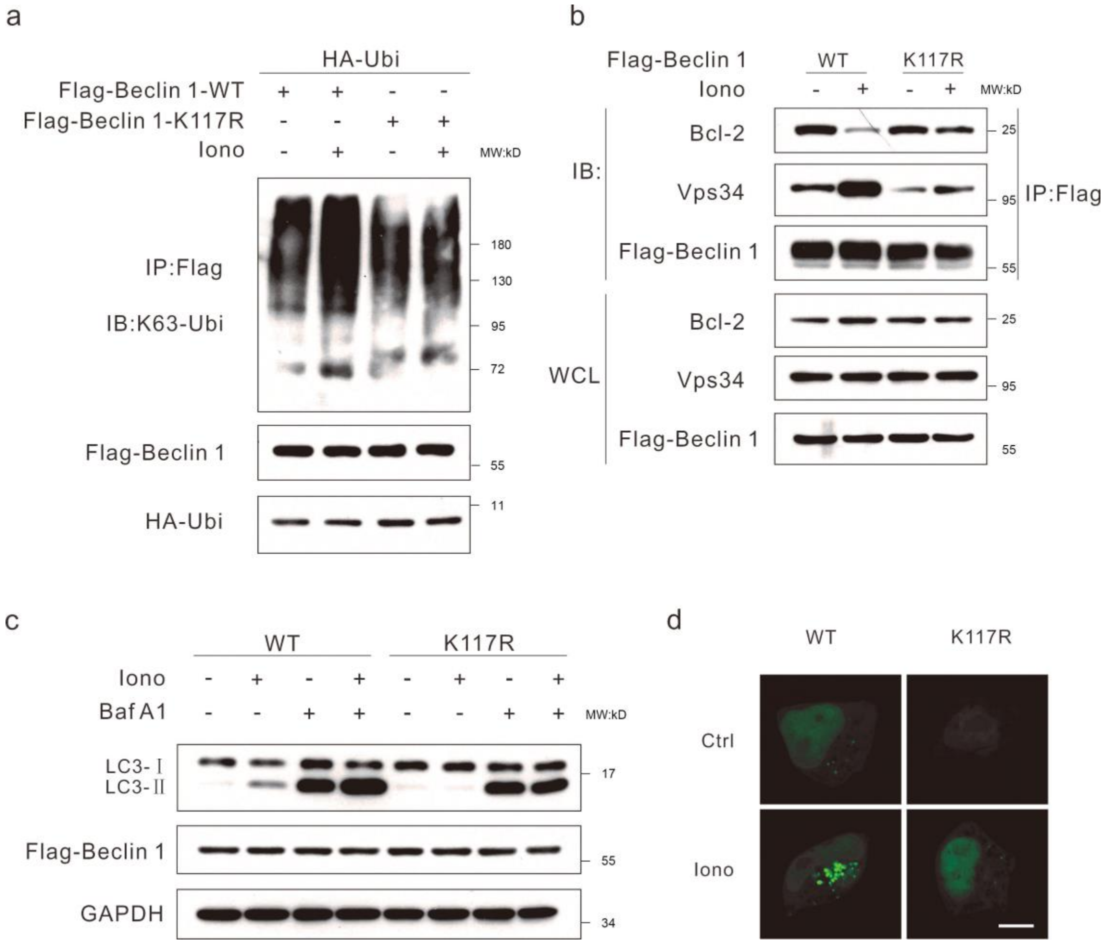

(a) The K63-ubiquitination of Beclin 1 induced by Ionomycin was occurred at Lys117 residue. HEK293T cells were transfected for 24 h with the indicated plasmids, incubated for 24 h with 6 μM ionomycin, and then treated with BafA1 for 4 h. The cells were lysed and incubated with an anti-Flag antibody prior to Western blot. The immunoprecipitates were subjected to Western blot analysis. WCL, the whole-cell lysate.

(b) The K117R mutant of Beclin 1 impaired the formation of Beclin 1 complex. HEK293T cells were transfected for 24 h with the indicated plasmids, incubated for 24 h with 6 μM ionomycin, and then treated with Baf A1 for 4 h. The cells were lysed and incubated with an anti-Flag antibody prior to Western blot. The immunoprecipitates were subjected to Western blot analysis. WCL, the whole-cell lysate.

(c) The K117 residue of Beclin 1 was required for the Ionomycin-induced autophagy. HEK293T cells were transfected for 24 h with the indicated plasmids, incubated for 24 h with 6 μM

ionomycin, and then treated with Baf A1 for 4 h. The cell lysates were then analyzed by Western blot using the indicated antibody.

(d) The K117 residue of Beclin 1 was required for the Ionomycin-induced autophagosome formation. The HeLa cells were transfected with plasmid encoding EYFP-LC3 and incubated with 6 μM ionomycin for 24 h and then subjected to an immunofluorescence analysis. Scale bars, 10 μm.

Supplementary Figure 8 Ionomycin treatment promotes degradation of inhibitor of differentiation proteins

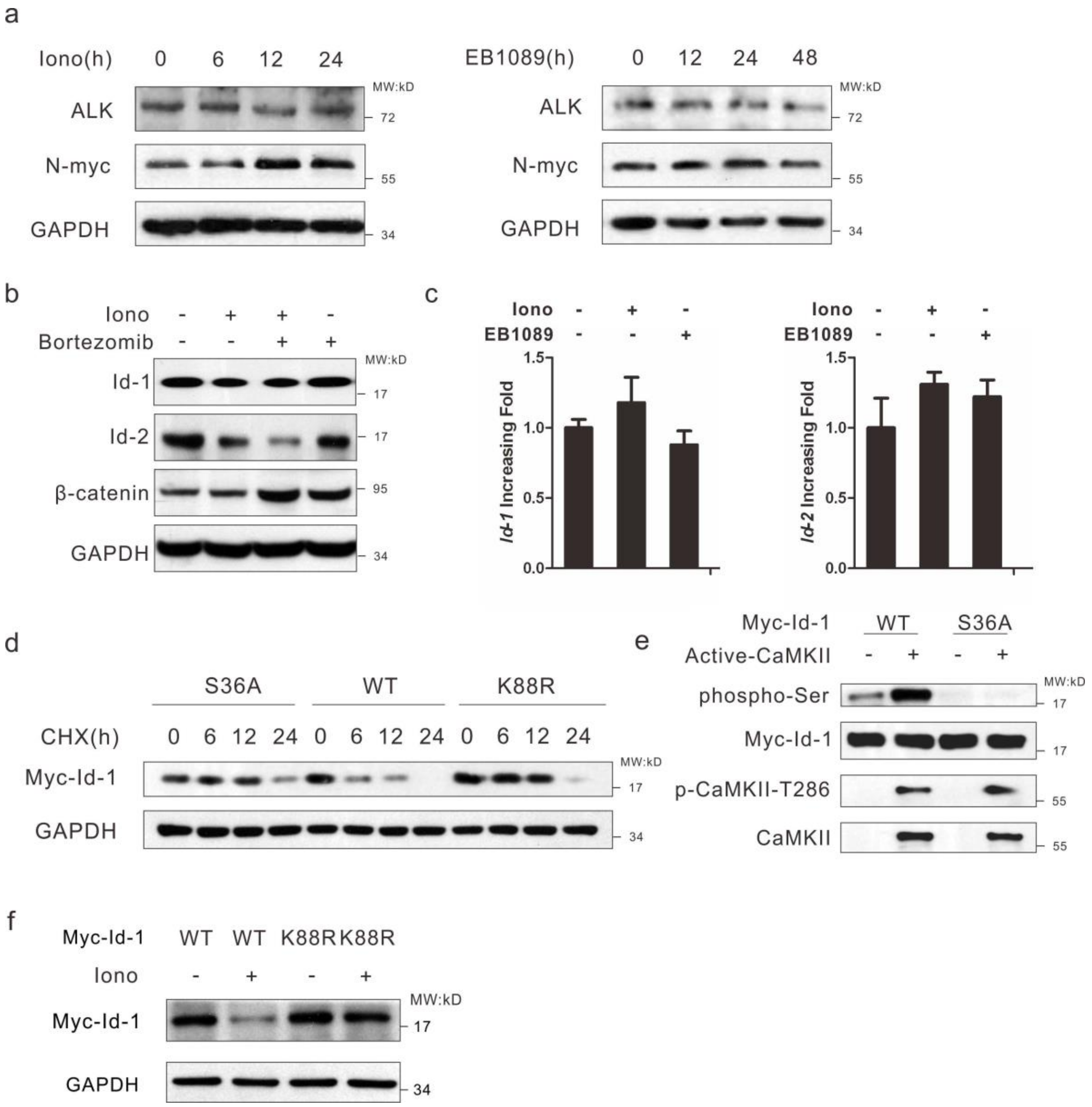

(a) Effect of ionomycin and EB1089 treatment on ALK and N-myc. SK-N-SH cells were treated with 6 μM ionomycin or 100 nM EB1089 for the indicated periods. The whole-cell lysates were analyzed by immunoblotting.

(b) The degradation of Id-1 and Id-2 cannot be blocked by a proteasome inhibitor bortezomib following ionomycin treatment. SK-N-SH cells were untreated or treated with 6 μM ionomycin for 24 h and then incubated for 4 h in the presence or absence of 1 μM bortezomib. The total-cell extracts were subjected to Western blot using the indicated antibodies.

(c) Ionomycin and EB1089 regulate the abundance of Id-1 and Id-2 at the post-transcriptional level. SK-N-SH cells were untreated or treated with 6μM ionomycin or 100 nM EB1089 for 24h.

The expression levels of *Id-1* and *Id-2* mRNA were detected by real-time RT-PCR. The error bars represent the standard deviations (SD) calculated from three parallel experiments.

(d) Id-1 K88R or S36A mutant proteins was more stable. 293T cells were transiently transfected with plasmids encoding Myc-Id-1-WT, S36A or K88R for 24h and then treated with 20μM CHX for the indicated time. The whole-cell extracts were subjected to western blotting using the indicated antibodies.

(e) CaMKII phosphorylated Id-1 *in vitro*. HEK293T cells transfected with Myc-Id-1 WT/S36A were treated with 6μM ionomycin, then lysed and the Id-1 proteins were precipitated by using the anti-Myc-tag antibody. The immunoprecipitate was incubated with recombinant active CaMKII kinase, and in vitro kinase assays were performed. Reaction products were run on SDS-PAGE and subjected to Western Blot.

(f) K88R mutant Id-1 was more stable. SK-N-SH cells were transiently transfected with plasmids encoding Myc-Id-1-WT or K88R for 24h and then treated with 6μM ionomycin for 24h. The whole-cell extracts were subjected to western blotting using the indicated antibodies.

Supplementary Figure 9 The effect of CaMKII on the differentiation of neuroblastoma cells.

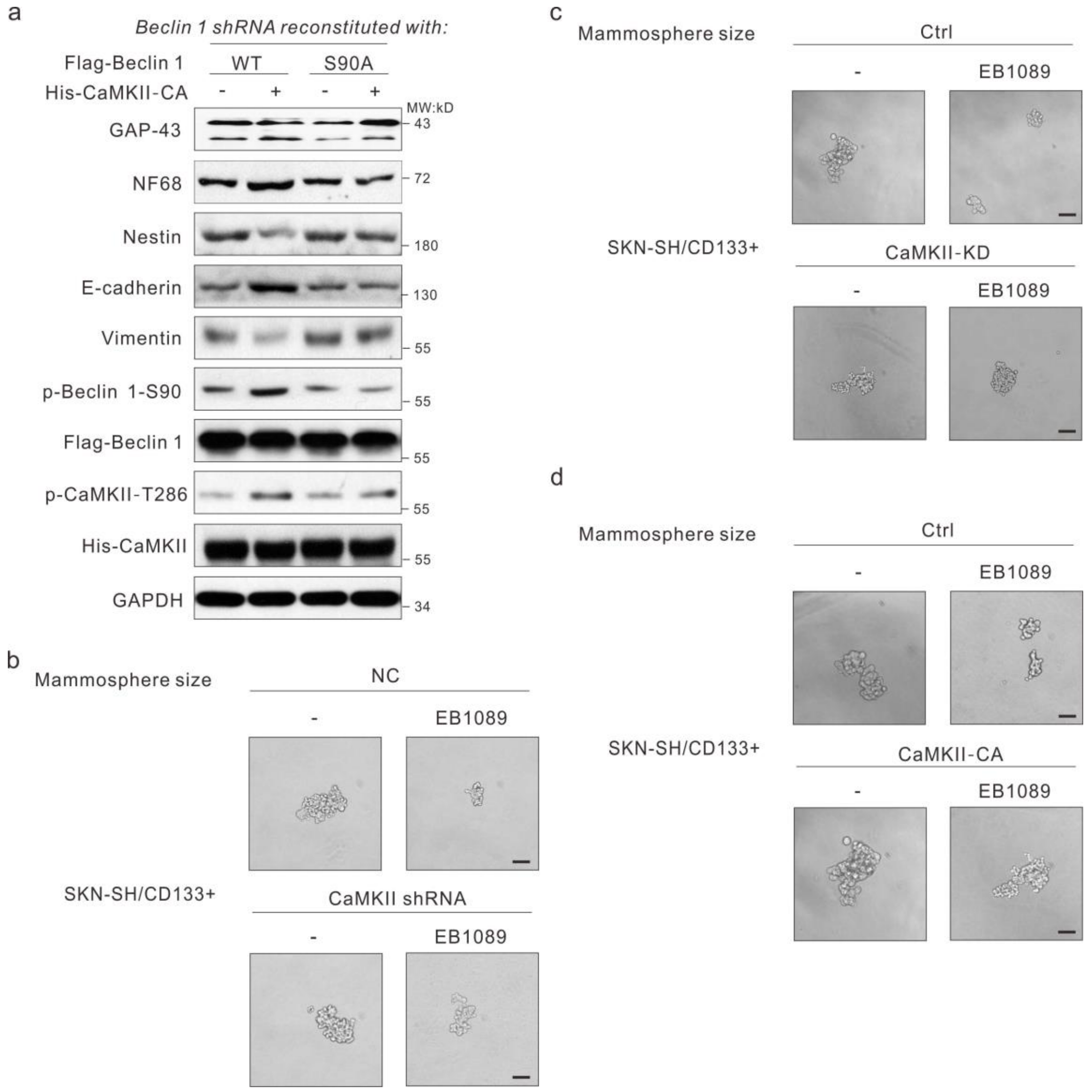

(a) Phosphorylation of Beclin 1-S90 by activated CaMKII is required for EB1089-induced differentiation in neuroblastoma cells. HEK293T cells were transfected with the indicated plasmids and then treated with 100 nM EB1089 for 24 h. The whole-cell extracts were subjected to Western blotting using the indicated antibodies.

(b) CD133 expression profiles were analyzed by flow cytometry in SK-N-SH cells transfected with a negative control or CaMKII shRNA. The CD133-positive cells were cultured in neurosphere-forming conditions. Neurospheres were incubated with 100 nM EB1089 for 7 days. Scale bars, 100 µm.

(c) CD133 expression profiles were analyzed by flow cytometry in SK-N-SH cells transfected with a control vector or plasmids encoding His-CaMKII-KD. The CD133-positive cells were cultured in

Neurosphere-forming conditions. Neurospheres were incubated with 100 nM EB1089 for 7 days. Scale bars, 100 µm.

(d) CD133 expression profiles were analyzed by flow cytometry in SK-N-SH cells transfected with a control vector or plasmids encoding His-CaMKII-CA. The CD133-positive cells were cultured in neurosphere-forming conditions. Neurospheres were incubated with 100 nM EB1089 for 7 days. Scale bars, 100 µm.

Supplementary Figure 10 The effect of phosphorylation of Beclin 1-S90 on neuroblastoma cell differentiation.

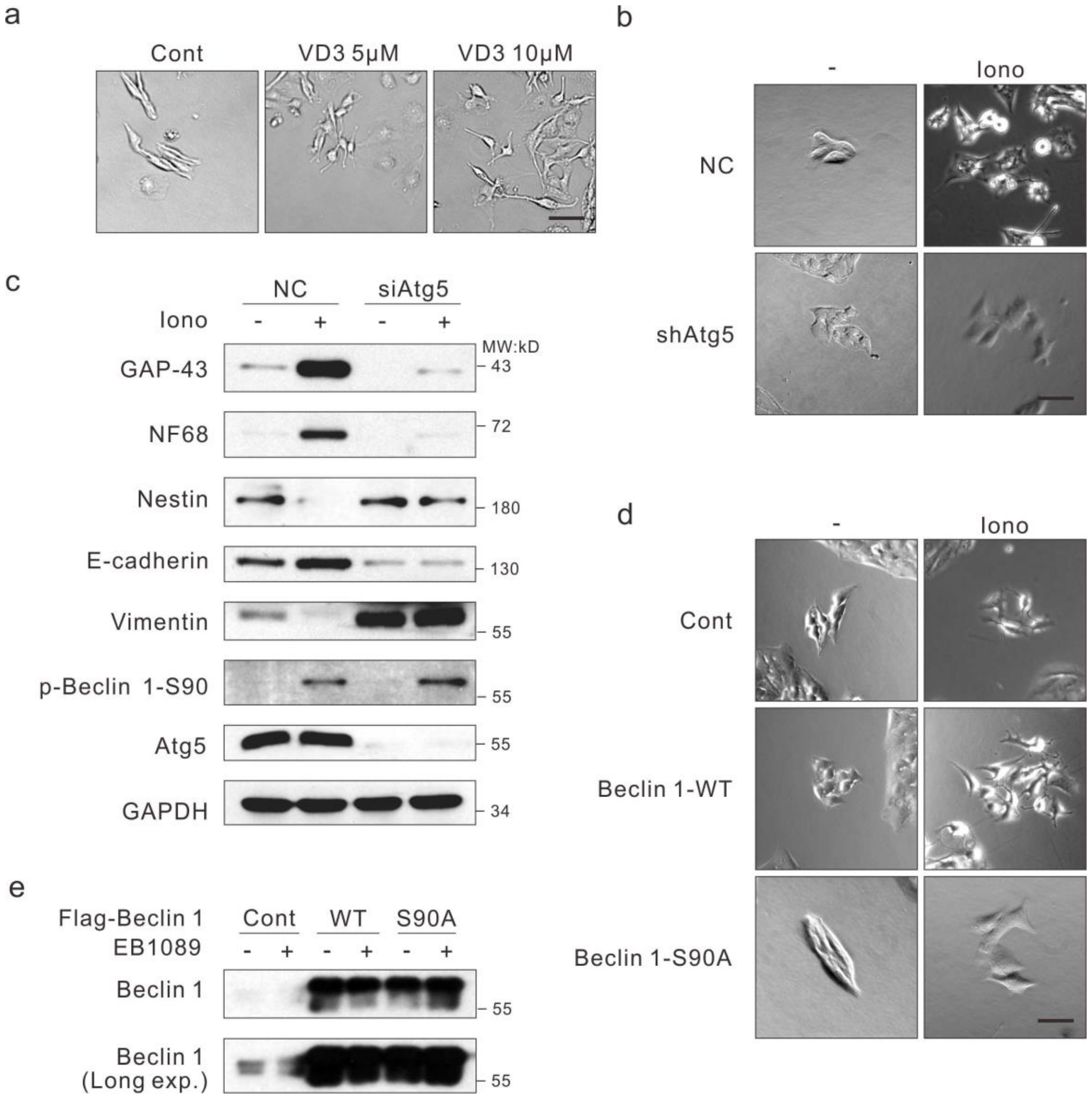

(a) Vitamin D3 regulates neuroblastoma cell differentiation. SK-N-SH cells were treated with 5/10 μM Vitamin D3 for 7 days and then observed using fluorescence microscopy. Scale bars, 30 μm.

(b) Autophagy is essential in ionomycin-induced autophagy. SK-N-SH cells were infected with lentivirus vectors encoding shRNA against Atg5 or a negative control, treated with 1.5 μM ionomycin for 7 days, and then observed using fluorescence microscopy. Scale bars, 30 μm.

(c) Autophagy is necessary for the cell differentiation induced by ionomycin. SK-N-SH cells were transiently transfected with NC, Atg5 siRNA and treated with 100 nM EB1089 for 24 h. The cell extracts were analyzed by Western blot.

(d) Phosphorylation of Beclin 1-S90 is required for ionomycin-induced differentiation in neuroblastoma cells. SK-N-SH cells stably expressing Vector, Beclin 1 (WT or S90A) treated with

1.5 μM ionomycin for 7 days, and then observed using fluorescence microscopy. Scale bars, 30 μm.

(e) The expression of Beclin 1 in 293T cells transfected with indicated plasmids. 293T cells were transiently transfected with Vector control, Flag-Beclin 1-WT or Flag-Beclin 1-S90A plasmids and treated with 100 nM EB1089 for 24h. The cell extracts were analyzed by Western blot.

Supplementary Figure 11 The effect of phosphorylation of Beclin 1-S90 by activated CaMKII on the proportion of CD133-positive cells.

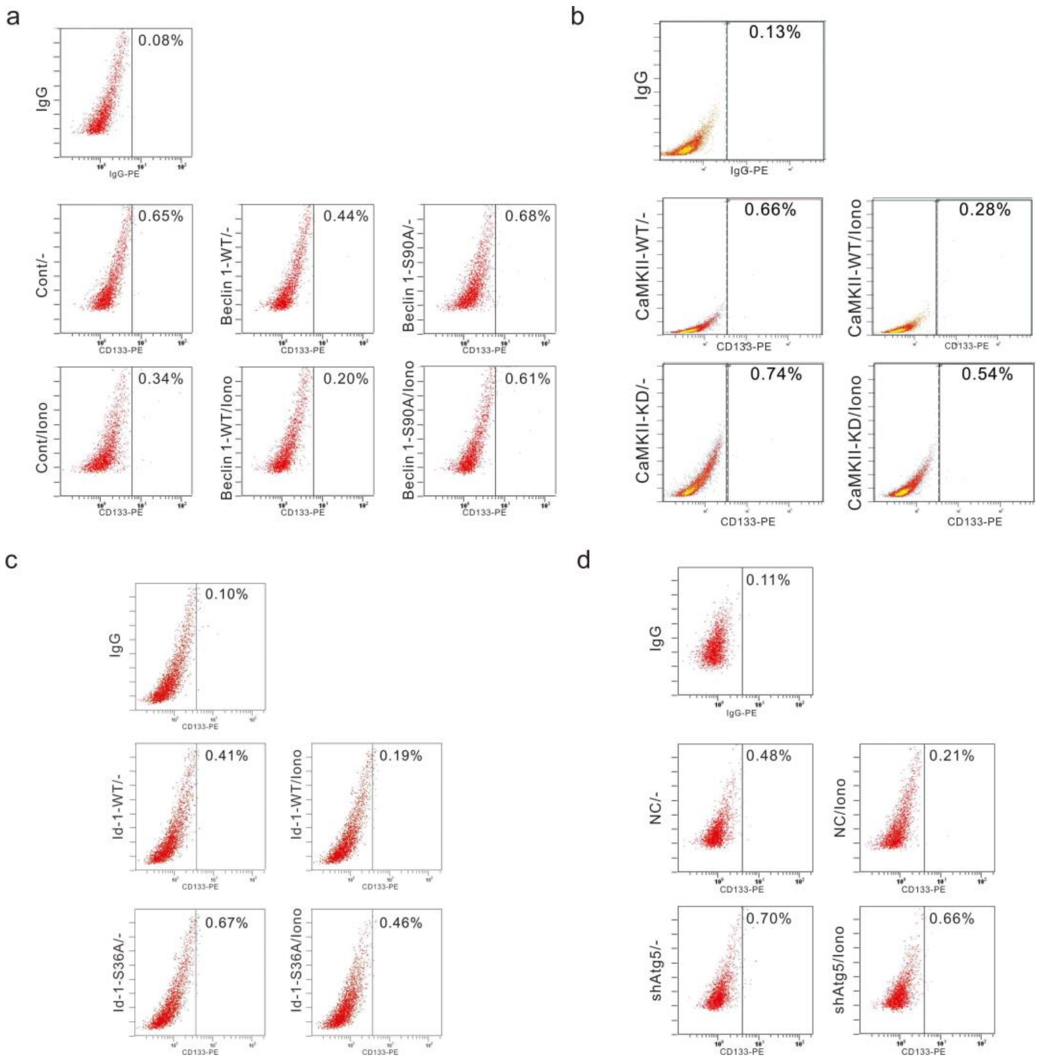

(a) SK-N-SH cells transfected with plasmids encoding Vector control, Flag-Beclin 1-WT or Flag-Beclin 1-S90A were untreated or treated with 6μM ionomycin for 24 h, and the CD133 expression profiles were analyzed by flow cytometry. CD133 fluorescence is depicted on the X axis, and the percentage of CD133-positive cells is shown in the right area of each plot.

(b) SK-N-SH cells transfected with a control vector or plasmids encoding His-CaMKII-KD were untreated or treated with 6μM ionomycin for 24 h, and the CD133 expression profiles were analyzed by flow cytometry. CD133 fluorescence is depicted on the X axis, and the percentage of CD133-positive cells is shown in the right area of each plot.

(c) SK-N-SH cells transfected with plasmids encoding Myc-Id1 or Myc-Id1-S36A were untreated or treated with 6μM ionomycin for 24 h, and the CD133 expression profiles were analyzed by flow

cytometry. CD133 fluorescence is depicted on the X axis, and the percentage of CD133-positive cells is shown in the right area of each plot.

(d) SK-N-SH cells transfected with plasmids encoding Vector control, shAtg5 were untreated or treated with 6μM ionomycin for 24 h, and the CD133 expression profiles were analyzed by flow cytometry. CD133 fluorescence is depicted on the X axis, and the percentage of CD133-positive cells is shown in the right area of each plot.

Supplementary Figure 12 The effect of phosphorylation of Beclin 1-S90 mediated by CaMKII on the proportion of CD133-positive cells.

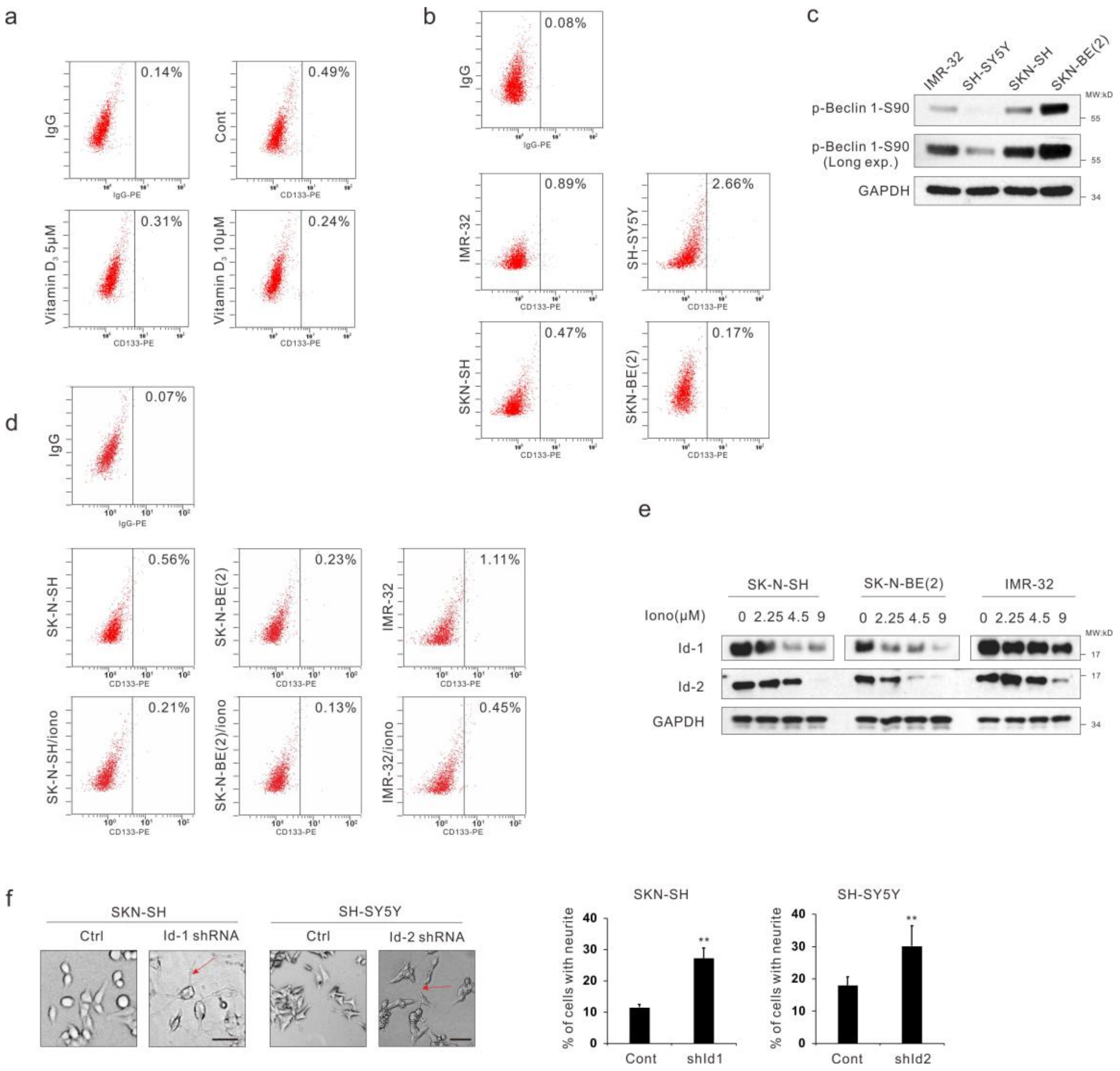

(a) SK-N-SH cells were untreated or treated with 5/10μM Vitamin D3 for 24 h, and the CD133 expression profiles were analyzed by flow cytometry. CD133 fluorescence is depicted on the X axis, and the percentage of CD133-positive cells is shown in the right area of each plot.

(b) Four kinds of nerublastoma cell lines were collected and the CD133 expression profiles were analyzed by flow cytometry. CD133 fluorescence is depicted on the X axis, and the percentage of CD133-positive cells is shown in the right area of each plot.

(c) The expression of Beclin 1-S90 phosphorylation in four kinds of nerublastoma cell lines.

(d) The proportion of CD133 positive cells in different kinds of neuroblastoma cell lines. Cells were treated with 6μM ionomycin for 24h, and the CD133 expression profiles were analyzed by flow cytometry. CD133 fluorescence is depicted on the X axis, and the percentage of

CD133-positive cells is shown in the right area of each plot.

(e) The degradation rates of Id-1/2 varied in different kinds of neuroblastoma cell lines. Cells were treated with indicated concentration of ionomycin for 24h. The whole-cell lysates were analyzed by immunoblotting.

(f) Id-1/Id-2 knockdown induced differentiation in neuroblastoma cells. Both cell lines were infected with lentivirus vectors encoding shRNA against Id-1/Id-2 or a negative control, then observed using fluorescence microscopy. Values were shown as the means ± SD of 5 random areas. ***P*<0.01, student's t-test. Scale bars, 30 μm.

Supplementary Figure 13 Full scans of uncropped blots presented in the main figures

Fig. 1c

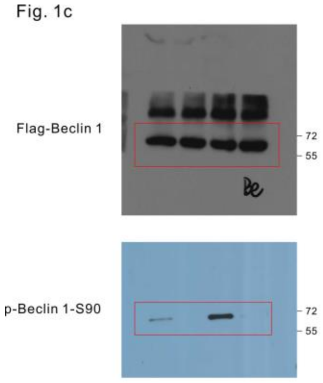

Fig. 1d

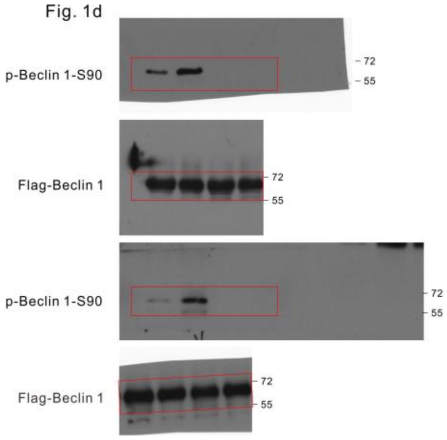

Fig. 1g

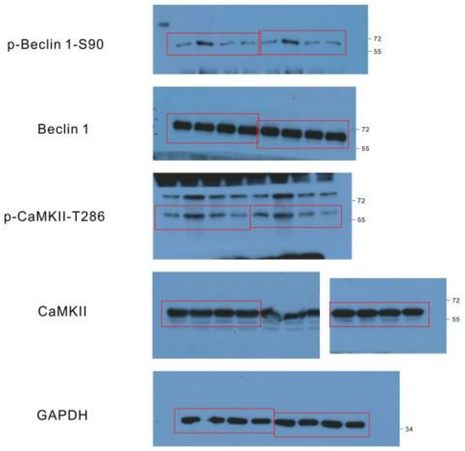

Fig. 1e

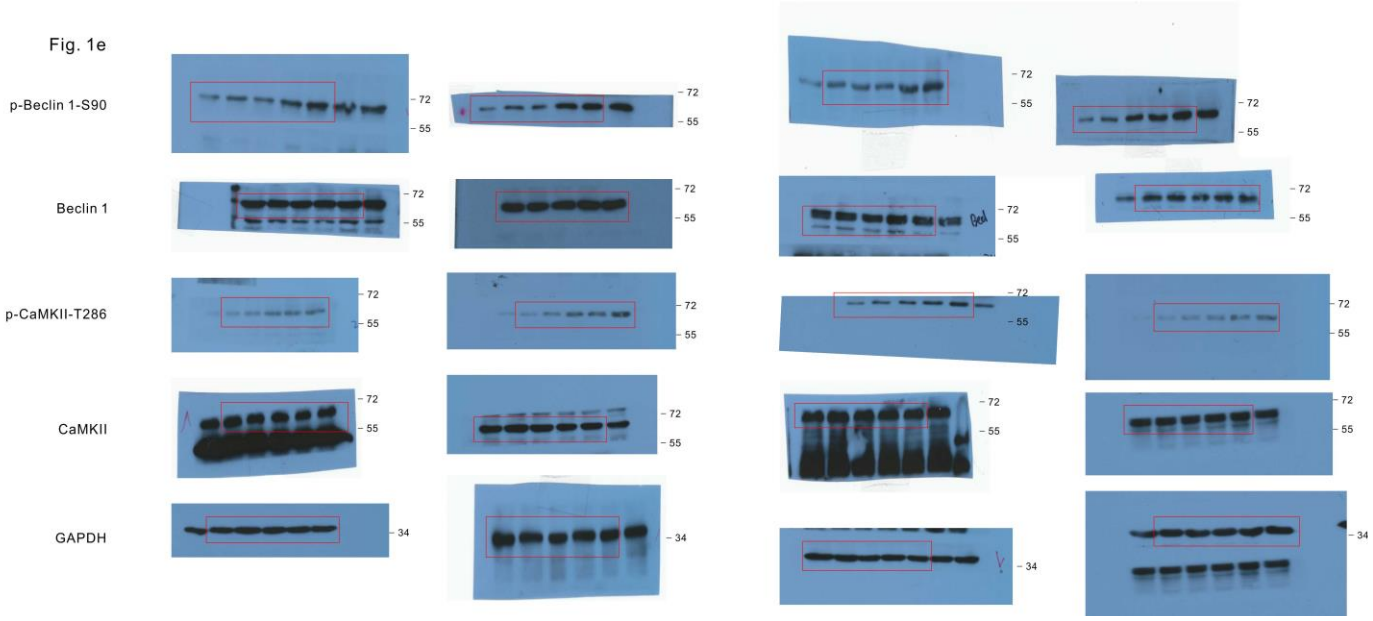

Supplementary Figure 13. continued

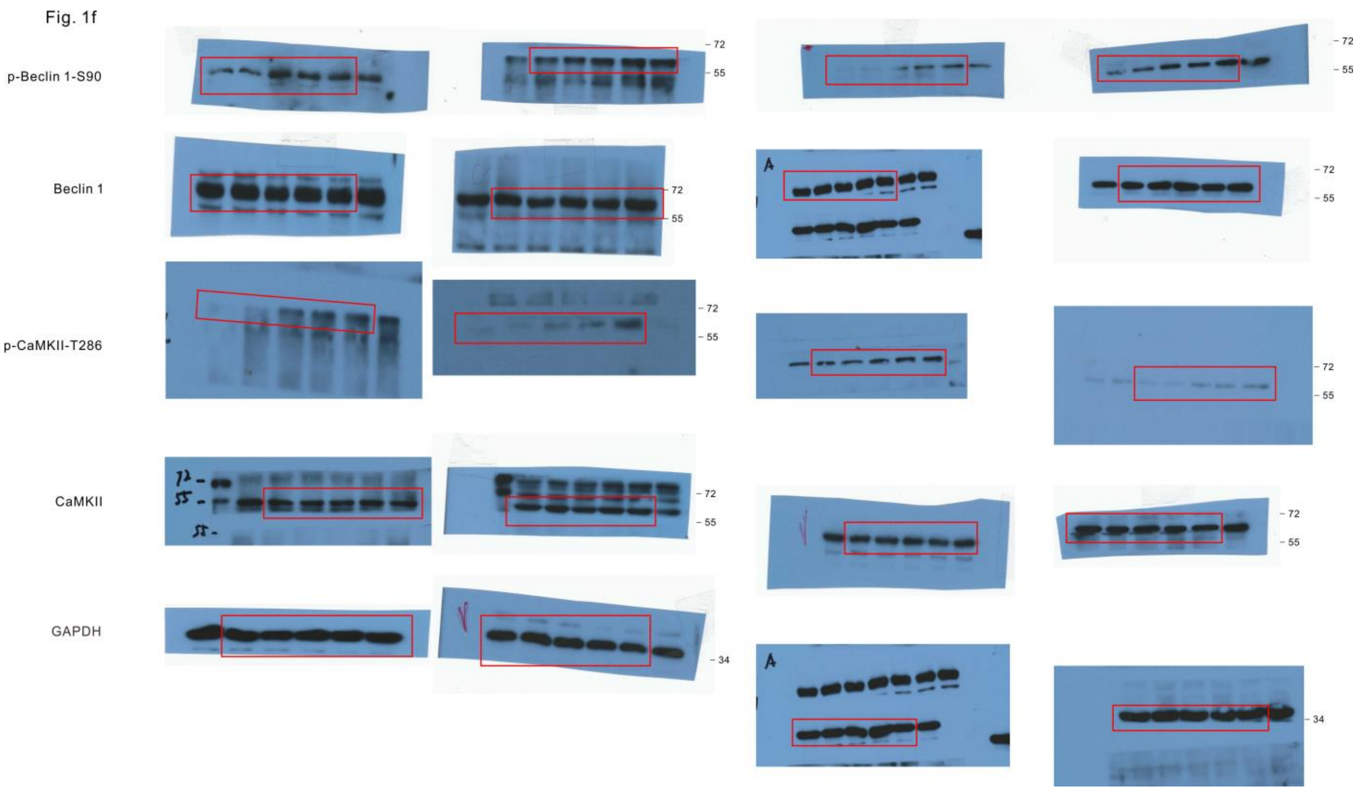

Supplementary Figure 13. continued

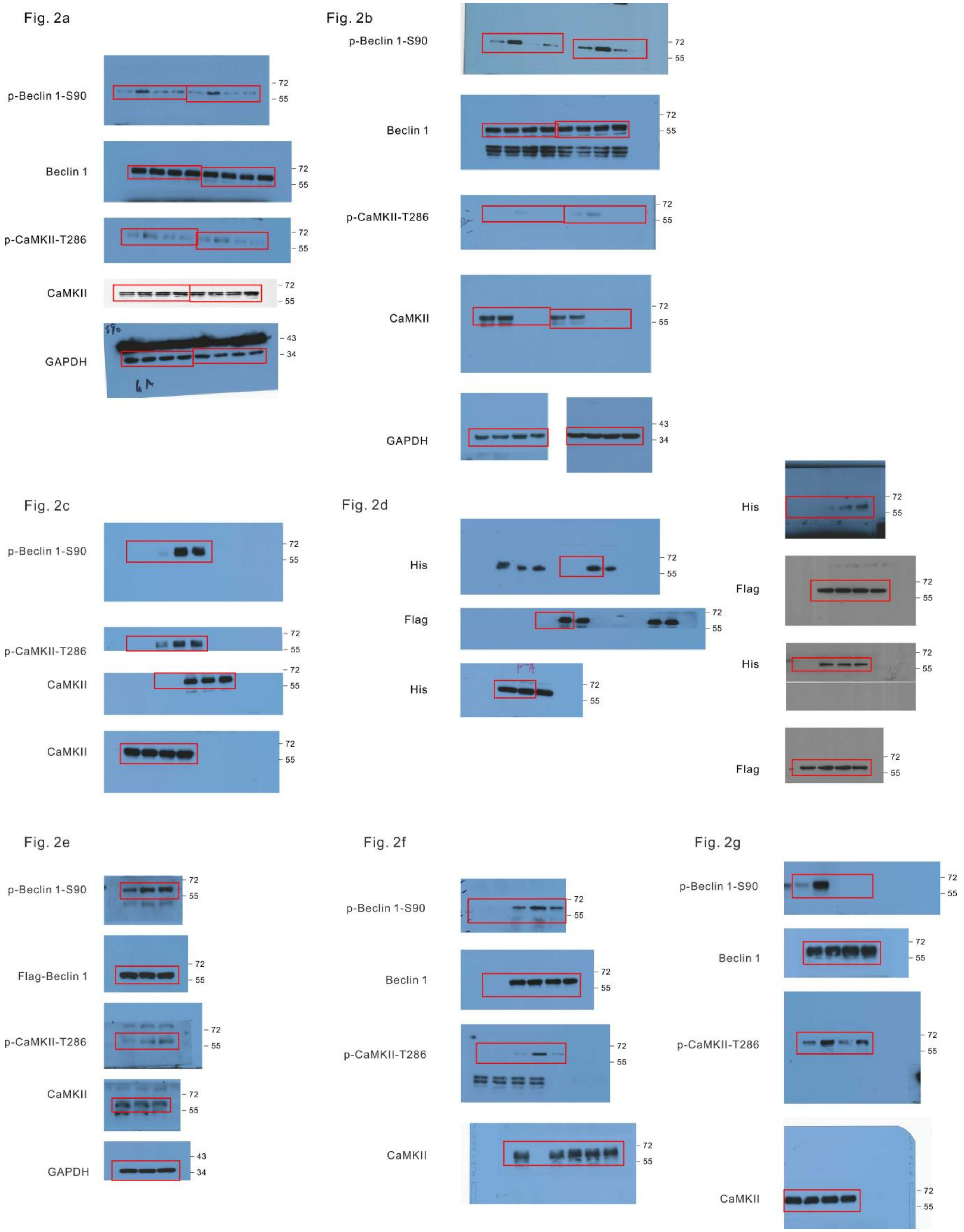

Supplementary Figure 13. continued

Fig. 3a

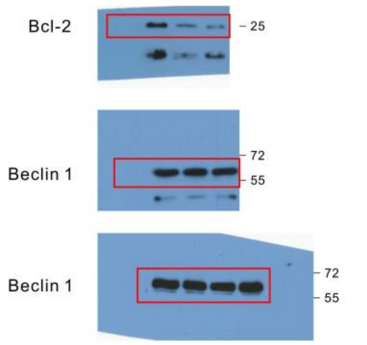

Fig. 3b

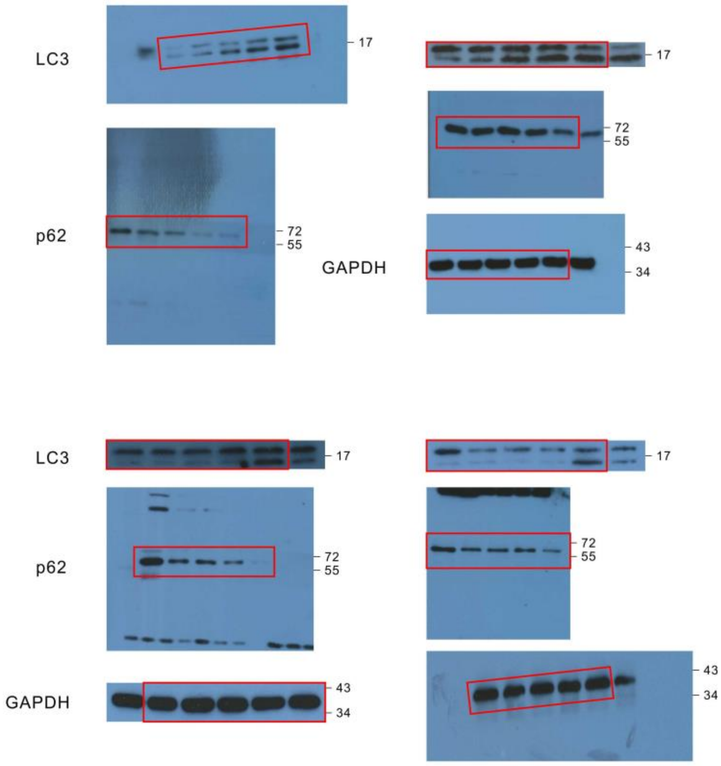

Fig. 3d/e

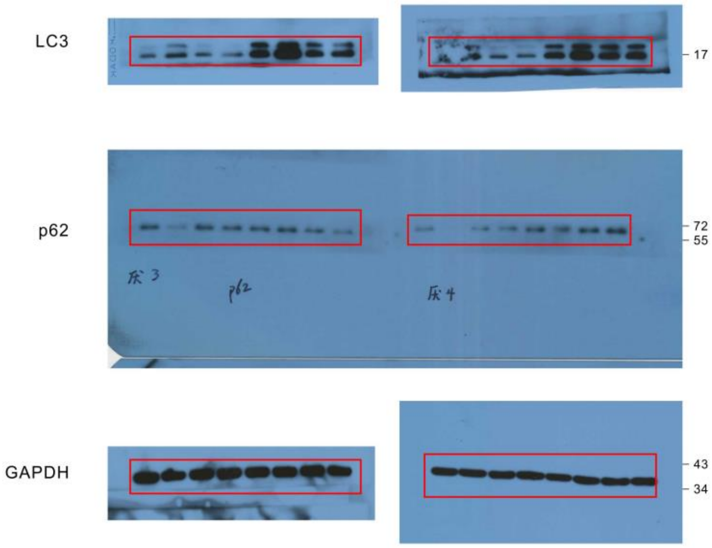

Fig. 3f

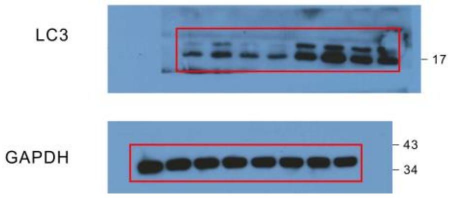

Supplementary Figure 13. continued

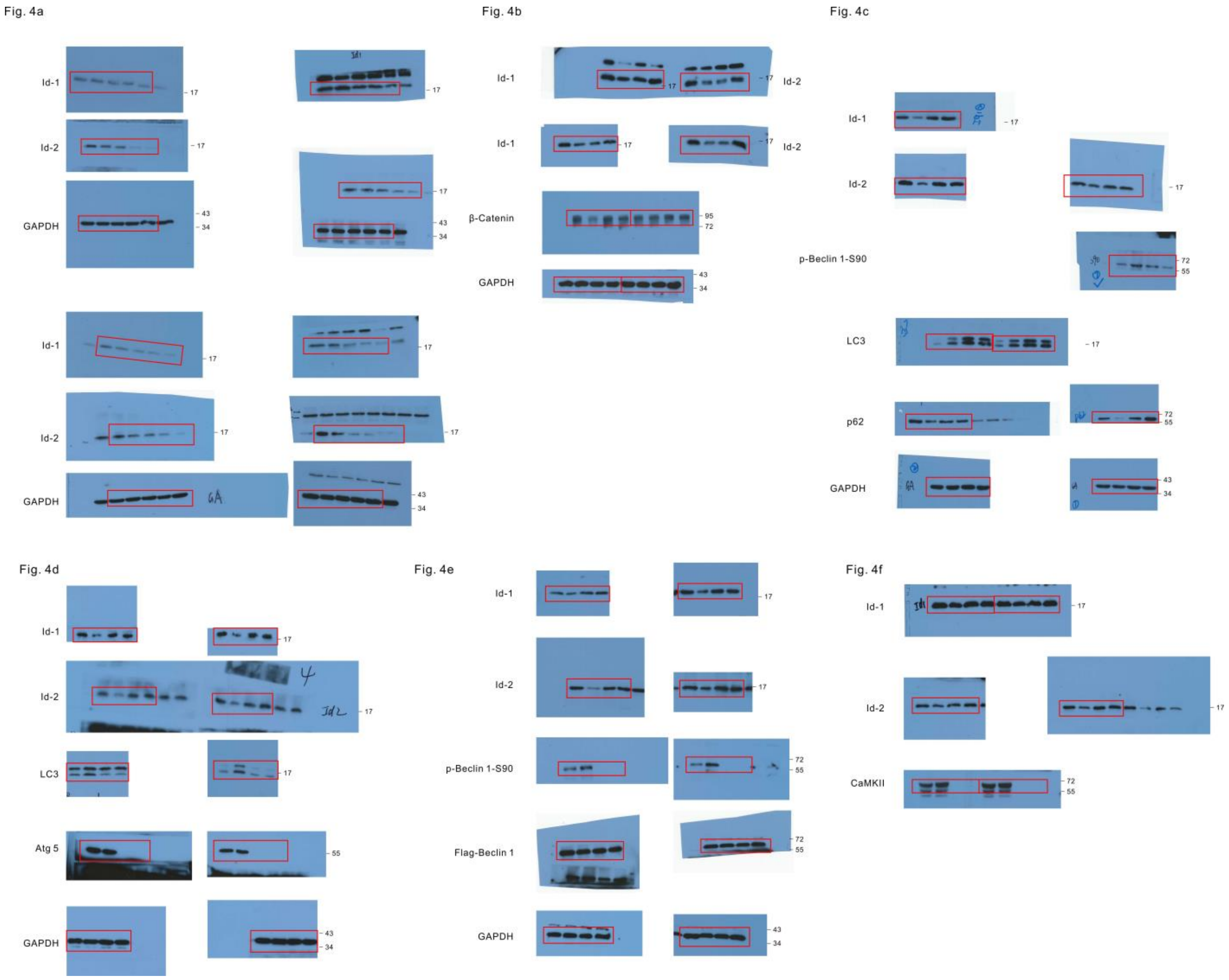

Supplementary Figure 13. continued

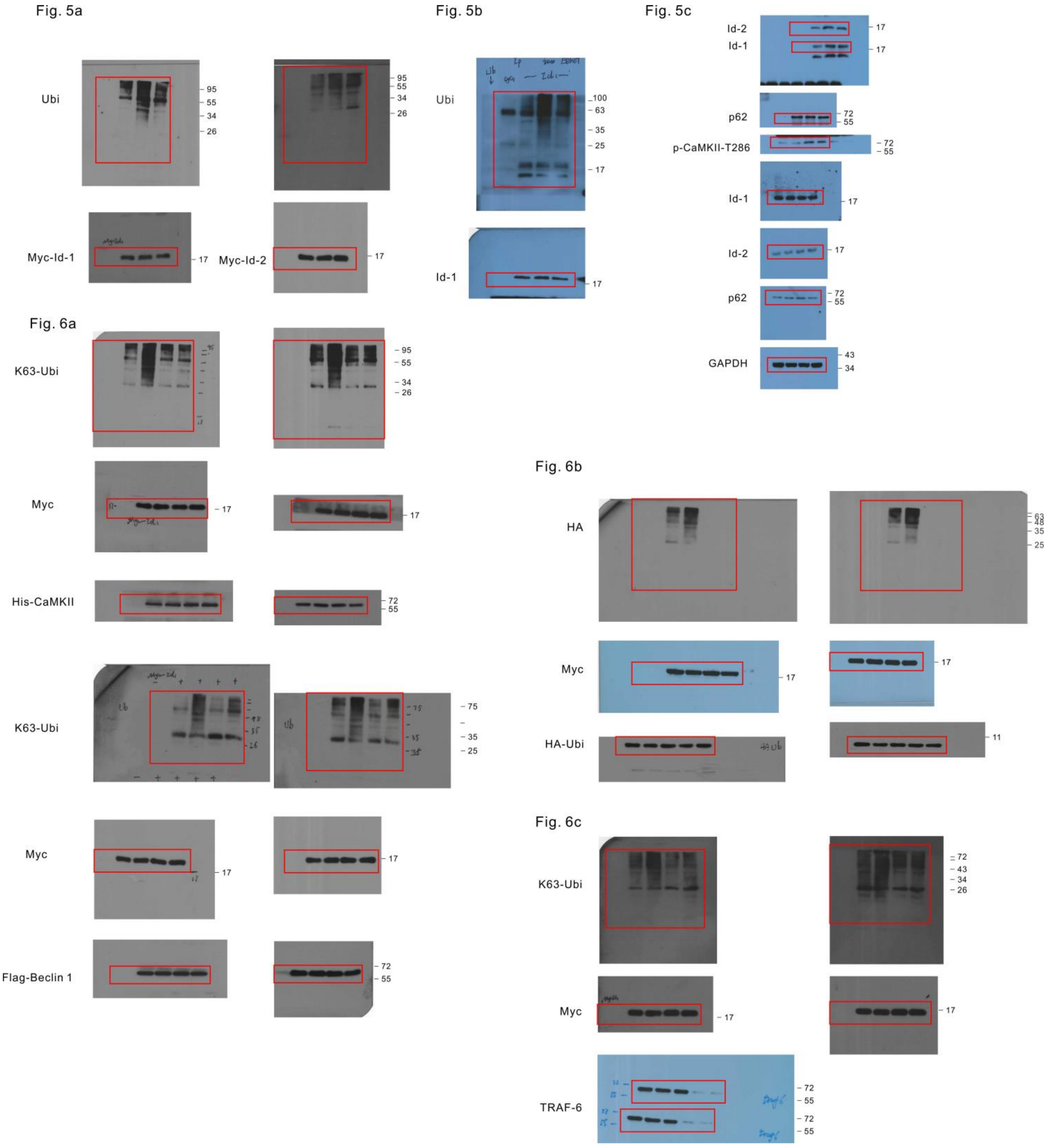

Supplementary Figure 13. continued

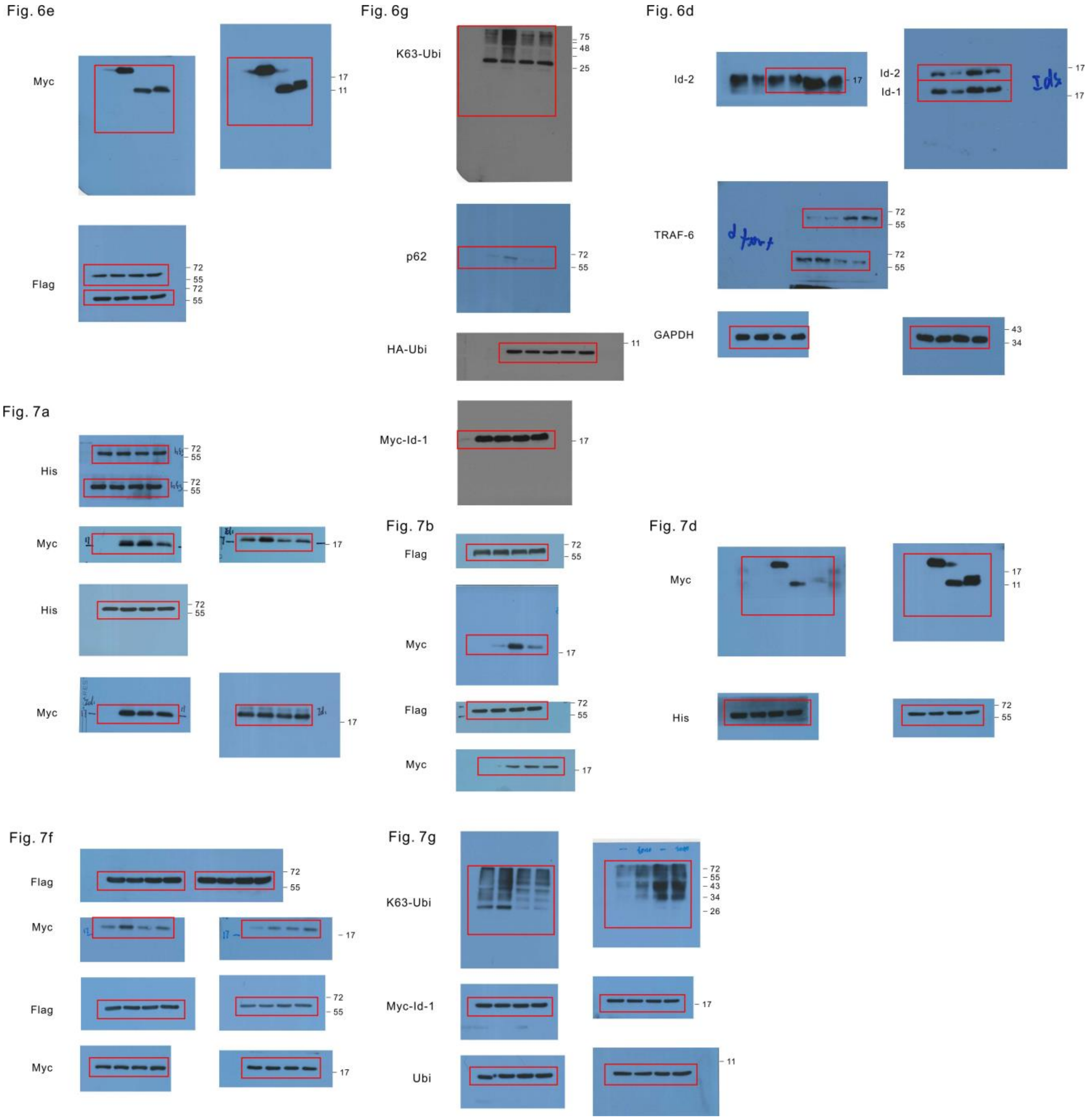

Supplementary Figure 13. continued

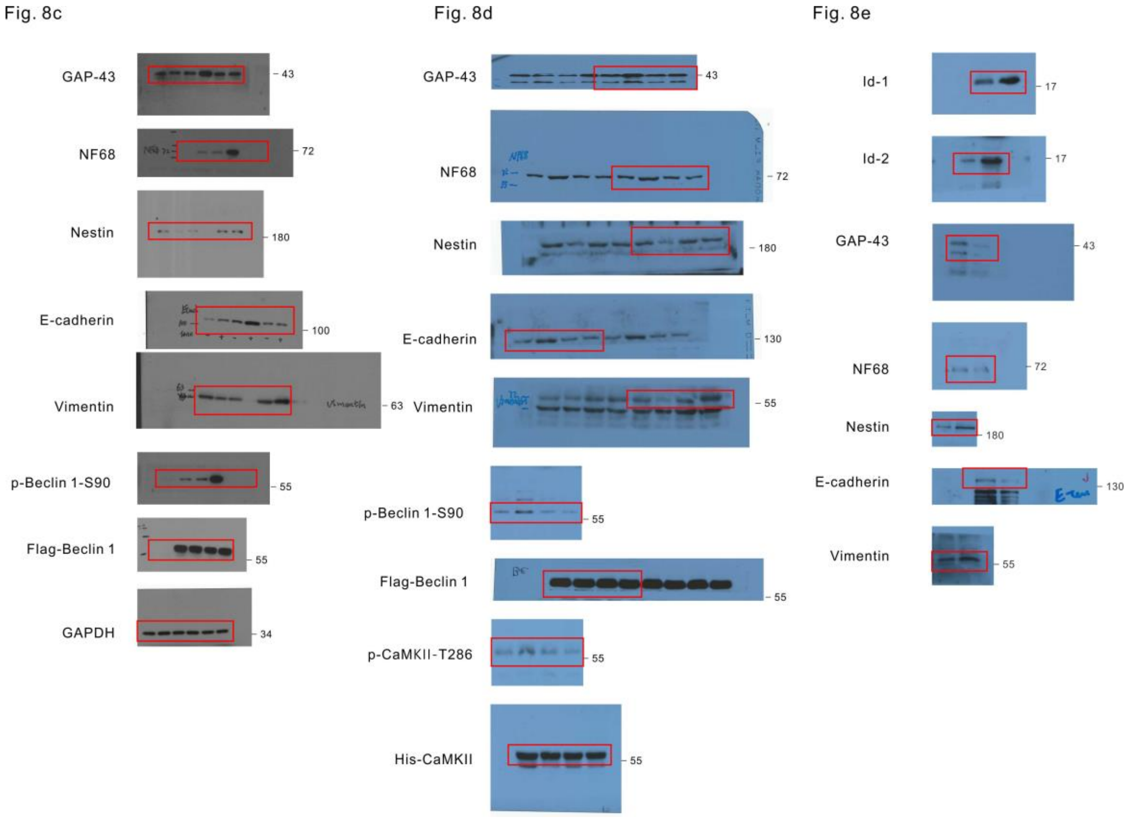

Supplementary Figure 14 Full scans of uncropped blots presented in the supplementary figures

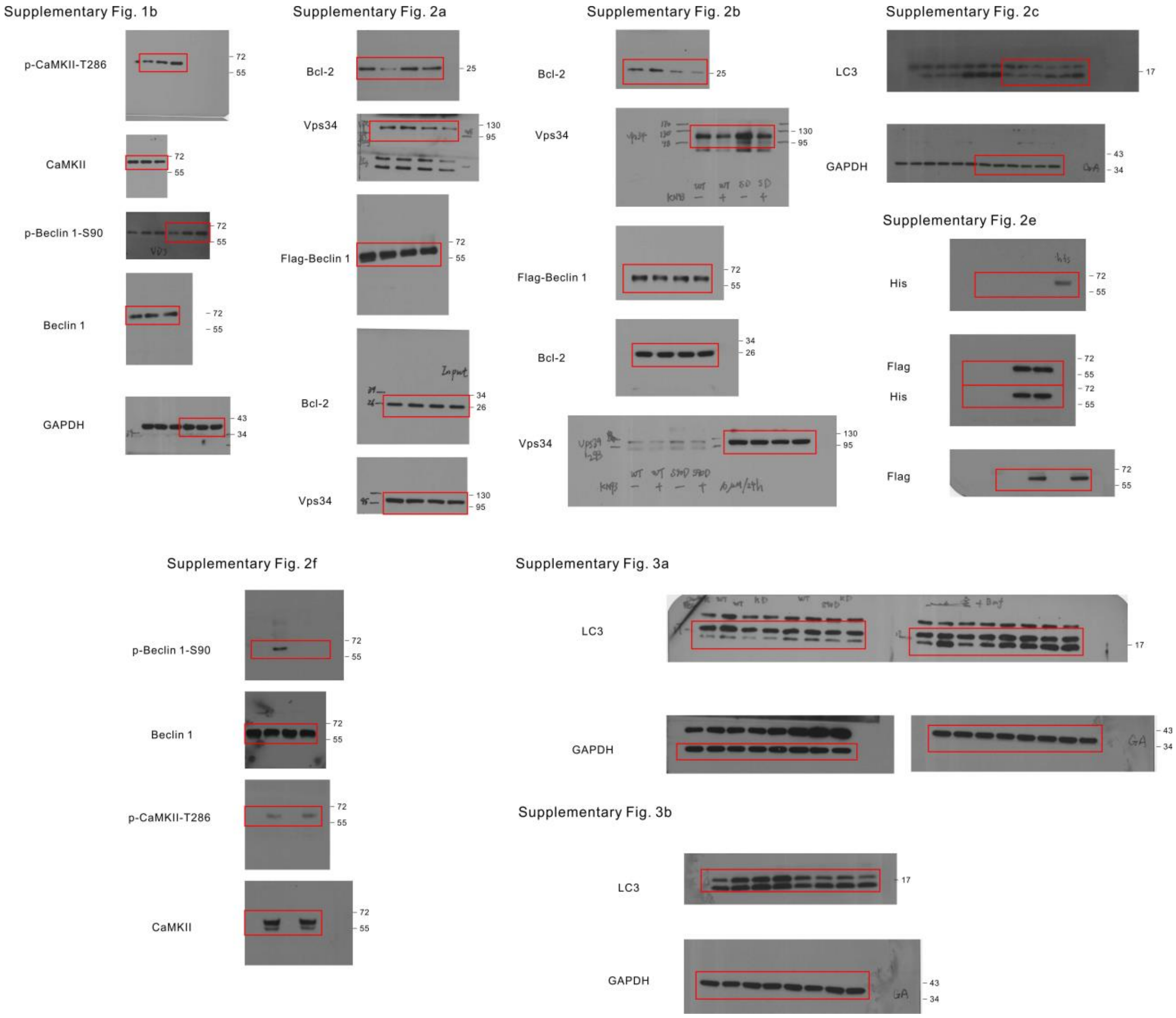

Supplementary Figure 14. Continued

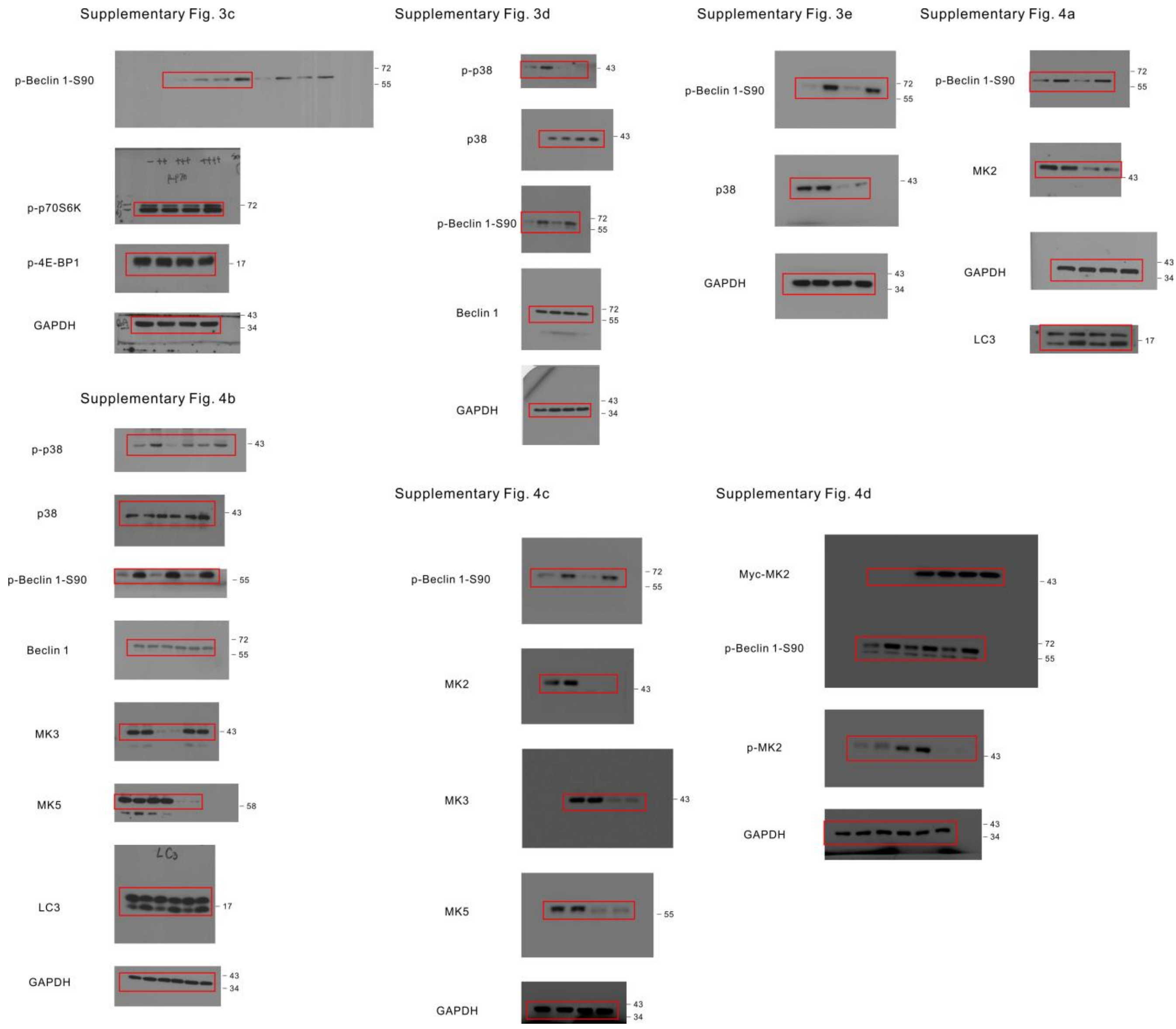

Supplementary Figure 14. continued

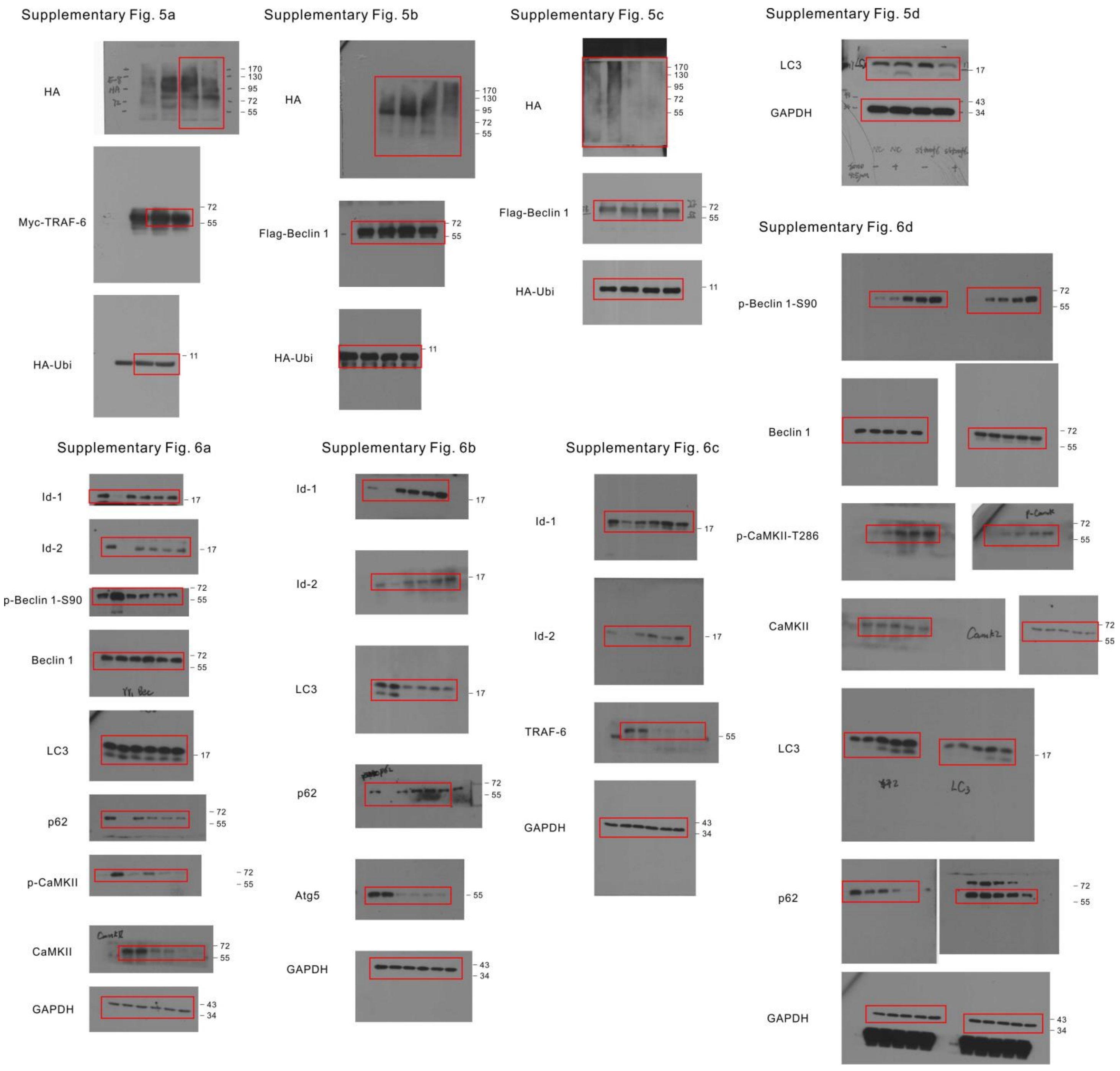

Supplementary Figure 14. Continued

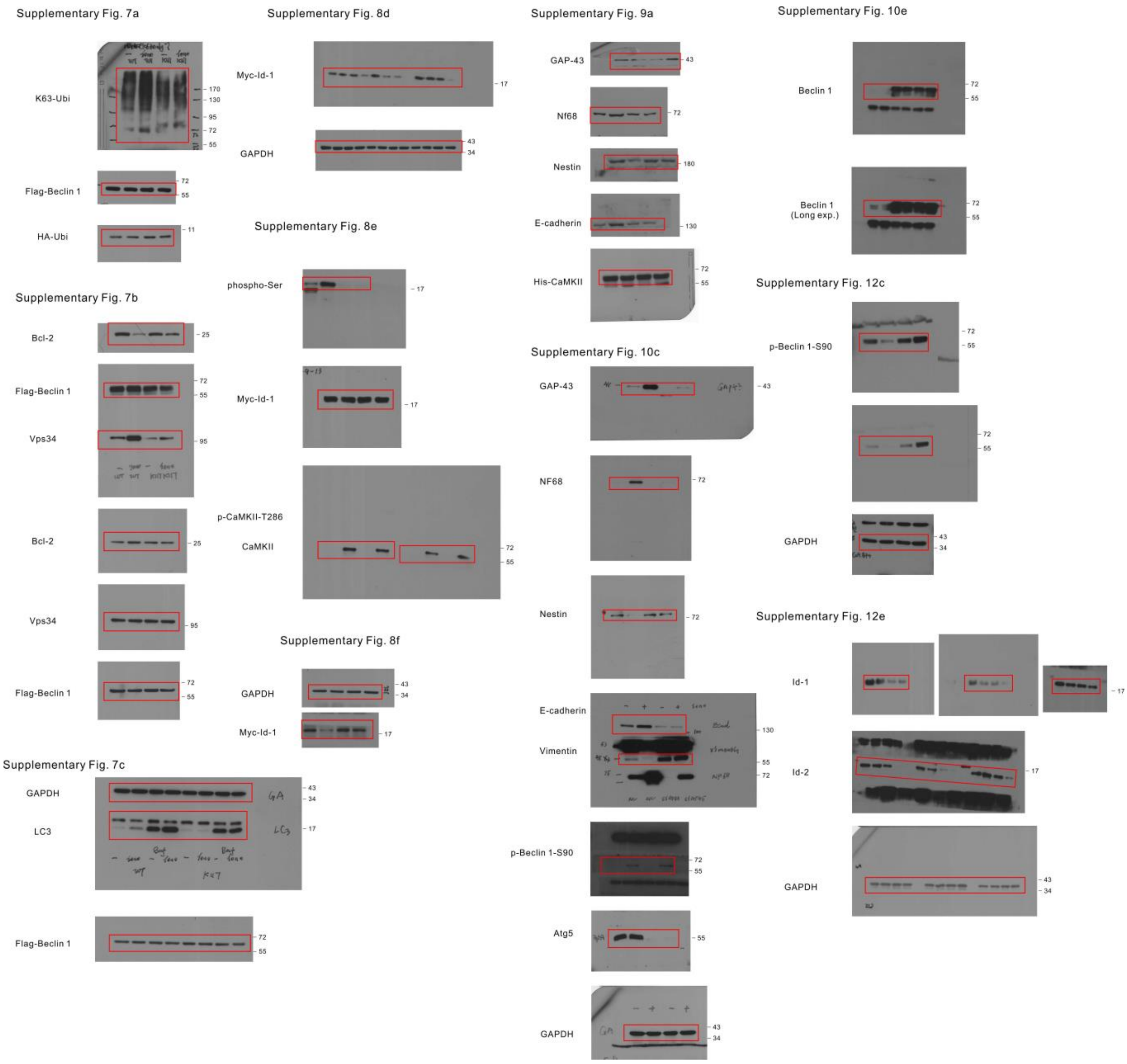

Supplement: Supplementary file 1 — Supplementary Information [file 41467_2017_1272_MOESM1_ESM.pdf]
